# Tissue Engineering for the Insertions of Tendons and Ligaments: An Overview of Electrospun Biomaterials and Structures

**DOI:** 10.3389/fbioe.2021.645544

**Published:** 2021-03-02

**Authors:** Alberto Sensini, Gabriele Massafra, Carlo Gotti, Andrea Zucchelli, Luca Cristofolini

**Affiliations:** ^1^Advanced Applications in Mechanical Engineering and Materials Technology – Interdepartmental Center for Industrial Research (CIRI-MAM), Alma Mater Studiorum-Università di Bologna, Bologna, Italy; ^2^Department of Industrial Engineering, Alma Mater Studiorum-Università di Bologna, Bologna, Italy; ^3^Health Sciences and Technologies – Interdepartmental Center for Industrial Research (CIRI-HST), Alma Mater Studiorum-Università di Bologna, Bologna, Italy

**Keywords:** electrospinning, enthesis, myotendinous junction, scaffolds, scaffolds biofabrication, cell cultures, *in vivo* tests, mechanical behavior

## Abstract

The musculoskeletal system is composed by hard and soft tissue. These tissues are characterized by a wide range of mechanical properties that cause a progressive transition from one to the other. These material gradients are mandatory to reduce stress concentrations at the junction site. Nature has answered to this topic developing optimized interfaces, which enable a physiological transmission of load in a wide area over the junction. The interfaces connecting tendons and ligaments to bones are called entheses, while the ones between tendons and muscles are named myotendinous junctions. Several injuries can affect muscles, bones, tendons, or ligaments, and they often occur at the junction sites. For this reason, the main aim of the innovative field of the interfacial tissue engineering is to produce scaffolds with biomaterial gradients and mechanical properties to guide the cell growth and differentiation. Among the several strategies explored to mimic these tissues, the electrospinning technique is one of the most promising, allowing to generate polymeric nanofibers similar to the musculoskeletal extracellular matrix. Thanks to its extreme versatility, electrospinning has allowed the production of sophisticated scaffolds suitable for the regeneration of both the entheses and the myotendinous junctions. The aim of this review is to analyze the most relevant studies that applied electrospinning to produce scaffolds for the regeneration of the enthesis and the myotendinous junction, giving a comprehensive overview on the progress made in the field, in particular focusing on the electrospinning strategies to produce these scaffolds and their mechanical, *in vitro*, and *in vivo* outcomes.

## Introduction

Tissue engineering is generally defined as “the creation (or formation) of new tissue for the therapeutic reconstruction of the human body, by the deliberate and controlled stimulation of selected target cells through a systematic combination of molecular and mechanical signals.” A material, in the form of a scaffold, is usually employed to provide a biomimetic morphology and mechanical properties to the tissue-engineered construct, facilitating the possible delivery of molecular and mechanical signals (Williams, 2009). Among the various techniques to produce scaffolds, electrospinning is getting increasing attention in the tissue engineering research field (Bosworth and Downes, 2011). Faithfully mimicking the extracellular matrix (ECM) of several tissues, such as the tendinous, ligamentous, muscular, and bony ones, it has demonstrated to allow the cell proliferation and growth (Baldino et al., 2015). Mainly in the last ten years, researchers have started to explore the possibility to develop dedicated strategies to regenerate multi-tissue structures (Haider et al., 2018). Focusing on the orthopedic side of the problem, this review will present a comprehensive overview on the electrospinning strategies adopted to produce scaffolds suitable for the regeneration of the tendon/ligament (T/L) to bone (enthesis) and the myotendinous junction (MTJ) interfaces. Section “METHODS: RESEARCH STRATEGY” clarifies the methods adopted to search and chose the papers analyzed in the review. In section “MAIN TISSUES OF THE MUSCULOSKELETAL SYSTEM,” after a brief overview on the main composition and properties of the musculoskeletal tissues, the enthesis and MTJ (section “Entheses and Myotendinous Junction”), structure (section “Structure”), and mechanical properties (section “Mechanical Properties”) are described. Then, an overview of the state of the art about of the surgical approaches to manage the injuries of this interfaces and the requirements for a scaffold for interfacial tissue engineering are reported (sections “TISSUE DAMAGE AND SURGICAL APPROACHES” and “REQUIREMENTS FOR A SCAFFOLD FOR INTERFACIAL TISSUE ENGINEERING”). In section “ELECTROSPINNING,” the principles of the electrospinning technique, the materials used, and the most relevant setups adopted are described. In section “RESULTS OF THE LITERATURE SEARCH,” “results of the literature search,” the most relevant works concerning electrospun scaffolds for the enthesis and the MTJ regeneration are analyzed and divided in increasing levels of hierarchical complexity (section “Overview of the Electrospun Scaffolds for the Enthesis and MTJ Regeneration”): simple mats (section “Simple Mats”), biphasic mats (section “Biphasic Mats”), multilayer mats (section “Multilayer Mats”), and composite and 3D structures (section “Composite and 3D Structures”). For each section, the works are listed describing the procedures to produce the scaffolds, followed by their morphological, *in vitro*, and *in vivo* mechanical performances. Finally, section “CONCLUSION AND FUTURE PERSPECTIVE” shows some conclusion and future perspectives of in the field.

## Methods: Research Strategy

A systematic search using the PubMed, Science Direct, and Google Scholar databases (from 1990 to September 2020) was carried out to find relevant papers in the electrospinning research field focusing on the regeneration of the enthesis and the MTJ. Moreover, to find additional papers possibly missed through the database searches, the list of citations from every paper was scanned. The titles, abstracts, and main texts of each work were examined, and only the papers truly relevant for this review were cited and incorporated. Inclusion criteria were manuscripts in English focusing on electrospun scaffolds for the regeneration of the enthesis and the MTJ. The following research strings were used to organize the different sections:

• For sections “MAIN TISSUES OF THE MUSCULOSKELETAL SYSTEM,” “TISSUE DAMAGE AND SURGICAL APPROACHES,” “REQUIREMENTS FOR A SCAFFOLD FOR INTERFACIAL TISSUE ENGINEERING,” and “ELECTROSPINNING,” to describe the natural tissue properties as well as the general guidelines for the interfacial tissue engineering, the most relevant papers concerning the following keywords were selected: tendon structure, ligament structure, bone structure, muscle structure, enthesis, tendon–bone interface, ligament–bone interface, muscle–tendon interface, myotendinous junction, tendon–bone injuries, ligament-bone injuries, myotendinous junction injuries, enthesis mechanical properties, myotendinous junction mechanical properties, and scaffolds for interface regeneration.
• To describe the several electrospinning strategies to mimic the enthesis and the MTJ (section “RESULTS OF THE LITERATURE SEARCH”), the following string was used: electrospinning AND tendon–bone OR enthesis OR tendon–bone healing OR ligament–bone OR ligament–bone healing/repair OR muscle–tendon junction OR myotendinous junction.

## Main Tissues of the Musculoskeletal System

The musculoskeletal system is composed by several tissues that work together ensuring the physiological movements. The main feature of these tissues is their hierarchical structures, organized in different levels of aggregation. The hard-structural component of this kinematic chain is the bone (Weiner and Wagner, 1998). It is mainly composed of collagen Type I fibrils doped with hydroxyapatite (Hap) nanocrystals (Rho et al., 1998). Tuning the spatial organization and percentages of these materials generates the cortical or cancellous bone (Rho et al., 1998). The cellular components of the bone are the osteocytes, the osteoblasts, and the osteoclasts (Florencio-Silva et al., 2015).

These natural joints are actuated by the muscles, mainly composed of water, proteins, salts, minerals, fat, and carbohydrates (Frontera and Ochala, 2015). The protein content consists of collagen (Types I, III, IV, and V) for their membranes (i.e., endomysium, perimysium, epimysium) (Light and Champion, 1984), while myosin, actin, and titin for their contractile units (i.e., sarcomeres) (Frontera and Ochala, 2015). Several thousands of sarcomeres produce a myofiber which is the structural contractile unit of muscles, composed of a single, polynucleated cell called myocyte (Frontera and Ochala, 2015). Bundles of myofibers form a muscle fascicle that is surrounded by the perimysium (Light and Champion, 1984). Then groups of fascicles generate the whole muscle belly.

Other key features of the musculoskeletal tissue are tendons and ligaments (T/L). T/L transmit the force between muscles and bones (tendons) and guarantee the physiological joint alignment (ligaments) (Arruda et al., 2006; Baldino et al., 2015). They are composed of water, collagen Type I, elastin, and proteoglycans (Murphy et al., 2016). The basic unit of T/L is the tropocollagen molecules that aggregate, producing the collagen fibril (Wang, 2006). The assembly of several fibrils produces substructures of increasing complexity (i.e., sub-fascicles, fascicles, tertiary fiber bundle) up to the whole T/L. These subunits are surrounded by collagen membranes (endotenon/endoligament) which, externally to the whole T/L, are named epitenon/epiligament (Kastelic et al., 1978; Kannus, 2000; Wang, 2006). The cellular component consisting of fibroblasts (ligaments) or tenocytes (tendons) is arranged in rows between the collagen fibers (Murphy et al., 2016; Santos et al., 2017).

Such different tissues are connected to each other with dedicated and fine-tuned junctions, fundamental to guaranteeing the physiological transfer of loads reducing the stress concentrations between them. Nature has answered to this issue by adopting progressive gradients of ECM organization/mineralization passing from one tissue to another (Baldino et al., 2015). These optimized interfaces are also able to drive the phenotype changes of the cellular component. In particular, the interface between the T/L to bone tissue is called enthesis, while the one between the tendon and muscle tissue is named myotendinous junction (MTJ) (Z. Paxton et al., 2012).

### Entheses and Myotendinous Junction

#### Structure

**FIGURE 1:**
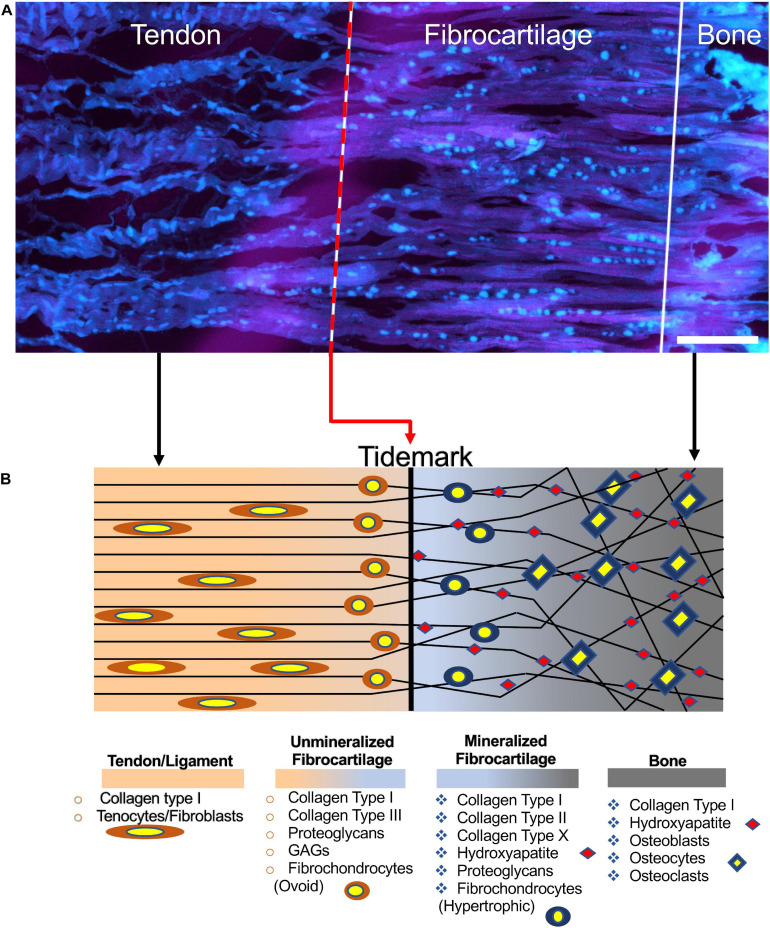
Structure of the enthesis. **(A)** Enthesis cryocut section of a porcine Achilles tendon was stained for cells using SYTO^®^ 13. Cells are depicted cyan (scale bar = 150 μm). Image adapted from Kuntz et al. (2018) (reproduced with permission under the terms of the CC BY 4.0 license. Copyright 2018, PLOS Publishing). **(B)** Graphical representation of the enthesis and its components.

The interface between T/L and bones, the enthesis, can widely vary depending on the anatomical sites and structures involved. However, two main enthesis categories are recognized as the most relevant: the fibrous enthesis (indirect) and the fibrocartilaginous enthesis (direct). In the fibrous enthesis, tendons and ligaments are connected through acute angles to bones with Sharpey’s fibers (8–25 μm) (Genin et al., 2009), collagen fibers extended directly from the periosteum (Yang and Temenoff, 2009). The fibrocartilaginous enthesis is characterized by a progressive mineralization gradient (Figure 1) organized in four zones (Yang and Temenoff, 2009): the T/L side, the unmineralized fibrocartilage, the mineralized fibrocartilage, and the bone side. The T/L tissue suffers progressive loss in anisotropy, increasing the mineralization content. The unmineralized fibrocartilage contains collagen (Types I, II, III, X, IX) as well as proteoglycans (mainly aggrecans) with associated chondroitin 4- and 6-sulfate glycosaminoglycans (GAGs) (Font Tellado et al., 2015). In this zone, the collagen fibrils increase their randomicity, while fibroblasts and tenocytes are replaced by ovoid-shaped aligned fibrochondrocytes (Yang and Temenoff, 2009). The boundary section between the unmineralized and mineralized fibrocartilage is called tidemark (Apostolakos et al., 2014). Then the mineralized fibrocartilage is found and continues up to the bone tissue. In this zone, it is possible to observe hypertrophic chondrocytes surrounded by collagen Types II and X and aggrecans (Baldino et al., 2015). In the fibrocartilage, the mean diameter of fibers ranges approximately 10–20 μm (Rossetti et al., 2017). Finally, the bone tissue is found. The enthesis is generally 500 μm thick along the T/L to bone junction (Rossetti et al., 2017).

**FIGURE 2:**
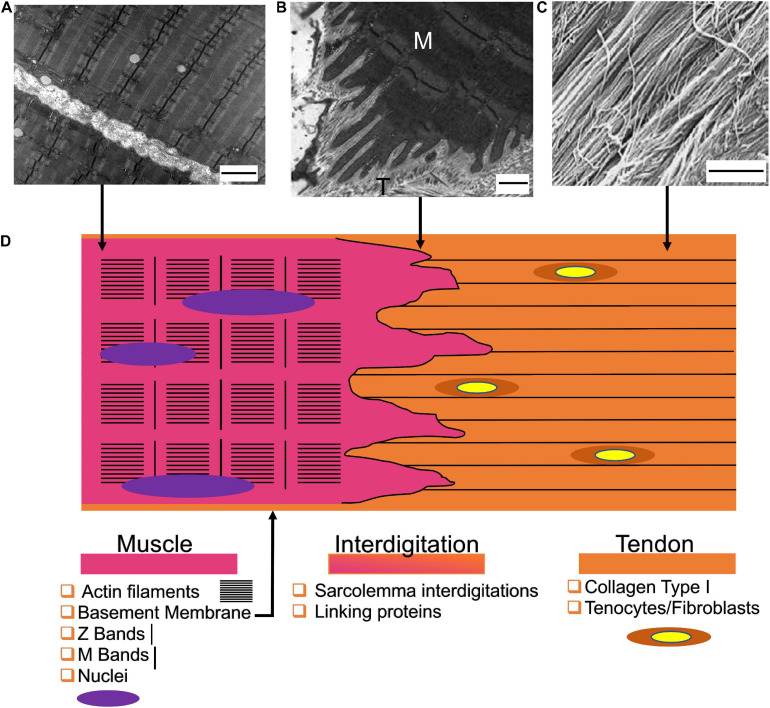
Structure of the myotendinous junction. **(A)** Transmission electron microscopy image of myofibrils (scale bar = 2 μm) (reproduced under CC0 1.0 Universal Public Domain Dedication. Author: Louisa Howard). **(B)** Transmission electron microscopy of the MTJ of the rat sternomastoid muscle (M = muscle side; T = Tendon side) (scale bar = 0.5 μm) adapted from Jacob et al. (2019) (reproduced with permission reproduced with permission under the terms of the CC BY-NC 4.0 license. Copyright 2019, PAGEPress.). **(C)** View of the tendon fibers observed with SEM (scale bar = 1.8 μm) adapted from Moshiri and Oryan (2013) (reproduced with permission under the terms of the CC BY 4.0 license. Copyright 2013, OMICS Publishing Group). **(D)** Graphical representation of the MTJ and its components.

The MTJ guarantees a gradual transition between the stiff tendon and the softer muscle. Differently to the enthesis, the MTJ connects each other a mainly cellular tissue (muscles) to a prevalent ECM-based one (tendons). At the macroscale, it creates a network of overlap between the muscle and tendon tissues, increasing their interface area (Figure 2). From a micro- and nanometric point of view, the myofibers at the MTJ generate conical finger-like projections interdigitating the tendon ECM (Charvet et al., 2012). Each muscle projection is composed of an aligned network of actin filaments matched with actin-binding proteins, that, originating from the Z-bands, gives to the projections their conical shape and stiffness (VanDusen and Larkin, 2015). This intracellular matrix is connected to subsarcolemma and intramembrane focal adhesion protein complexes. This allows to anchor the muscle cytoskeleton to the tendon ECM. Several focal adhesion complexes are present at the MTJ, containing proteins such as talin, vinculin, and paxillin (Tidball et al., 1986; Turner, 2000). The adhesion complexes connect to the actin matrix of the sarcolemma projections (i.e., the transmembrane proteins α7 integrin and the dystrophin-associated glycoproteins) which join to the extracellular laminin, anchoring the muscle cytoskeletal proteins to the tendon ECM (VanDusen and Larkin, 2015).

#### Mechanical Properties

The main mechanical feature of natural tissues is their nonlinear properties (Fung, 1993; Murphy et al., 2016), which are strongly dependent on the degree of mineralization (Fung, 1993; Murphy et al., 2016). When a load is applied, the fibrils/myofibers are progressively stretched, from their resting crimped state up to the complete alignment in the linear region (Maganaris and Narici, 2005; Gotti et al., 2020a). Because of the mineral content, the bone behavior results more brittle and stiffer (Rho et al., 1998) compared with the muscle and T/L ones (Table 1). Considering these extensive ranges of mechanical properties, dedicated connections are fundamental to reducing the stress concentrations and guaranteeing a progressive tissue gradient. Moreover, since these interfaces have small surfaces, it is difficult to measure their mechanical strength without considering partial contributions from the surrounding tissues. For this reason, very few works have tried to study the mechanics of the enthesis and the MTJ (Table 1).

**TABLE 1:** Ranges of mechanical properties of the different musculoskeletal tissues.

Tissue	Stiffness (MPa)	Failure stress (MPa)	Failure strain (%)	References
**Bone**				
Cortical (human)	14000–21800	82.9–150.6	1.0–3.1	Reilly and Burstein, 1975; Ashman et al., 1984; Mirzaali et al., 2016
Cancellous (human)	1.7–1624.4	0.06–20.8	0.49–26.8	Lindahl, 1976; Morgan et al., 2003; Matsuura et al., 2008
Tendon (human)	99.6–926	7.3–116	9–55.5	Noyes et al., 1984; Bechtold et al., 1994; Johnson et al., 1994
Ligament (human)	23–724	1–82.8	1.3–164.2	Siegler and Schneck, 1988; Neumann et al., 1992; Pintar et al., 1992; Savelberg et al., 1992
Muscle (human)	0.03–8	0.07–0.8	30–60	Lännergren, 1971; Halpern and Moss, 1976; Moss and Halpern, 1977; Kovanen et al., 1984; Lakie and Robson, 1988; Lieber et al., 1991; Wolff et al., 2006; Hoang et al., 2007; Lv et al., 2010; Kuthe and Uddanwadiker, 2016; Schleifenbaum et al., 2016
**Enthesis**	3100	2-49	1-61	Deymier et al., 2017; Li et al., 2017; Song et al., 2019; Reifenrath et al., 2020
Supraspinatus (rat)	3.1 ± 0.9	45.7 ± 3.4	4.3 ± 3.3	Deymier et al., 2017
Supraspinatus (rat)	-	33 ± 35	-	Deymier et al., 2019
Supraspinatus (rat)	20–80	8–20	-	Deymier et al., 2020
**MTJ**				
Diaphragm (pig)	0.28 ± 0.15	0.15 ± 0.02	122.4 ± 19.2	Ladd et al., 2011
Achilles tendon–triceps surae (pig)				Zhao et al., 2018
Distal	∼ 90	20–40	45–75	Zhao et al., 2018
Proximal	70–150	40–60	35–55	Zhao et al., 2018
Gastrocnemius (turkey)	744 ± 219	53.2 ± 12.9	8.6 ± 4.2	Azizi et al., 2009

## Tissue Damage and Surgical Approaches

Although the entheses dissipate the stress away from the interface, there are many cases of tear and wear (Benjamin et al., 2002). The injuries of the entheses generally occur at the rotator cuff, the anterior cruciate ligament (ACL), the Achilles tendon, and the medial collateral ligament (Derwin et al., 2018). The enthesis injuries often result in severe disability and may cause osteoarthritis, which is estimated to affect over 70% of people in the range of 55–78 years old (Boys et al., 2017). Moreover, even younger patients can be affected by acute or overuse sport injuries like tennis elbow and jumper’s knee (Calejo et al., 2019). There are approximately 2 million Achilles tendon sports related injuries in the world every year. Among them, over 250000 require surgical intervention (Baldino et al., 2016). The failure rates after surgeries for enthesis repair are extremely high (e.g., rotator cuff: 20–94%; ACL: 10–25%) (Po-Yee Lui et al., 2010).

Also, the MTJ has a great risk of stress concentration-related injuries (Speer et al., 1993). The injuries at the MTJ are classified in 3 degrees: (i) small lesions, (ii) partial, and (iii) complete tears (Palmer et al., 1999). In the first degree, the MTJ shows limited lesions and edemas which heal without any permanent consequences (Taneja et al., 2014). In the second degree, a partially ruptured junction is shown, causing pain, and recurrent injury may occur. These injuries are treated with a conservative approach that generally restores the muscle strength and its range of motion (Palmer et al., 1999). The surgical approach to manage the third degree (i.e., total rupture) depends on several factors such as the patient’s age, the rupture site, and the actual range of motion. A possible way to repair injured interfaces is provided by biological grafts (i.e., autografts, allografts, and xenografts). Due to the several limitations of the autografts (i.e., collateral lesions), allografts (risk of infections), xenografts (zoonosis), and the fast loss of mechanical properties, regeneration of these interfaces is still a challenge (Chen et al., 2009). Trying to improve the surgical performances, researchers focused on the production of synthetic scaffolds able to mimic the entheses and MTJ structure and mechanics. This particular branch of tissue engineering is named “interfacial tissue engineering” (Bonnevie and Mauck, 2018).

## Requirements for a Scaffold for Interfacial Tissue Engineering

To guarantee a biomimetic reproduction of the structure and mechanics of the target biological tissue, scaffolds have to meet some requirements, which are essential to promoting the healing:

1. Biocompatibility: scaffolds must be accepted by the human body and not considered as a foreign object. Cells have to recognize the scaffolds’ surface as biomimetic and adhere and proliferate on them. The poor biocompatibility can cause inflammatory processes, which can lead to infections or foreign-body rejections (Chen et al., 2009; O’Brien, 2011).
2. Biodegradability: scaffolds must be engineered to have a controlled degradation over time. This is mandatory to allow cells to replace scaffolds with new ECM (O’Brien, 2011). The degradation rate is fundamental: if it is too high, the mechanical competence is lost too quickly, while cells are unable to proliferate on scaffolds (Sung et al., 2004). Moreover the degradation process must not release toxic components causing inflammations (O’Brien, 2011).
3. Porosity: scaffolds must be porous to allow the cellular infiltration, proliferation (Loh and Choong, 2013) and the clearance of waste products from the scaffolds themselves (O’Brien, 2011).
4. Mechanical properties: the mechanical properties should be congruent with the site of implantation, protecting the interfaces from peaks of load until the ECM is under regeneration (Chen et al., 2009). In fact, the mechanical behavior of scaffolds is fundamental to speed up the cellular proliferation, differentiation, and the ECM production (Francois et al., 2015).
5. Morphology: scaffolds must follow the morphology of the different tissues that are going to temporarily replace. This implies the production of continuous gradients of fiber orientation, materials, and structures of the ECM (Zhang et al., 2012).
6. Mineralization (for enthesis only): T/L to bone entheses present a gradation in mineral content, which starts at the tidemark and intensifies along the mineralized fibrocartilage and cancellous bone tissue. A dedicated scaffold for the regeneration of these tissues needs to replicate this mineral gradient (Zhang et al., 2012).

## Electrospinning

Electrospinning allows to produce fibers with diameters from a few micrometers down to the nanoscale (Bosworth and Downes, 2011). In fact, electrospinning constituted a ground-breaking revolution in tissue engineering when researchers realized its ability to mimic the ECM driving the cellular proliferation and growth (Ramakrishna et al., 2005; Bosworth and Downes, 2011). The physical phenomenon starts when a target polymer (or blend), solved in a dedicated solvent system, is extruded by a metallic needle. To produce a high electrostatic field, the needle is charged with a positive voltage and placed at the opposite side of a metallic collector posed at ground potential. This setup causes the distribution of positive charges over the surface of the polymeric solution droplet, extruded by the needle (Haider et al., 2018). The positive charges are attracted to the ground collector, causing the formation of the so-called Taylor’s cone and the production of a fiber. During the flying phase, from the needle to the ground collector, the fibers are stretched, losing the solvents, reducing their diameter. Despite that electrospinning is theoretically a simple technique, it is influenced by several parameters which directly affect the resulting nanofibers (Haider et al., 2018). These parameters are grouped in three macro categories (Ramakrishna et al., 2005):

(1). Solution parameters: molecular weight of polymers involved, solution viscosity, surface tension, solution conductivity, dielectric constant, and boiling point of solvents.
(2). Process parameters: applied voltage, feed rate, collector shape and movement, diameter and structure of the needle, movement of the needle, number of needles, and needle-collector distance.
(3). Environmental parameters: relative humidity, temperature, and pressure.

**FIGURE 3:**
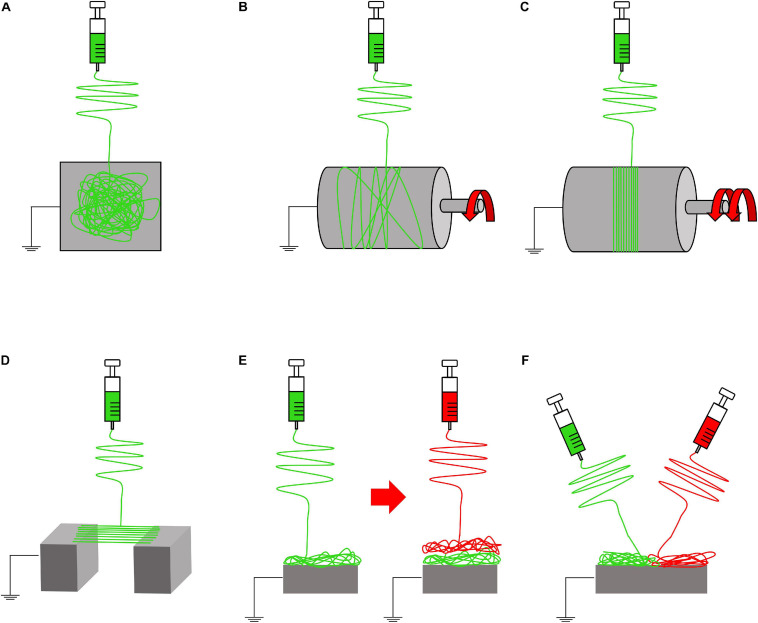
Electrospinning setups to produce nanofibrous mats. **(A)** Flat plate collector (random nanofibers). **(B)** Rotating drum collector (rotational speed < 8 m/s = random nanofibers). **(C)** Rotating drum collector (rotational speed ≥ 8 m/s = aligned nanofibers). **(D)** Gap collector (aligned nanofibers). **(E)** Multilayer electrospinning configuration. **(F)** Co-electrospinning setup.

**FIGURE 4:**
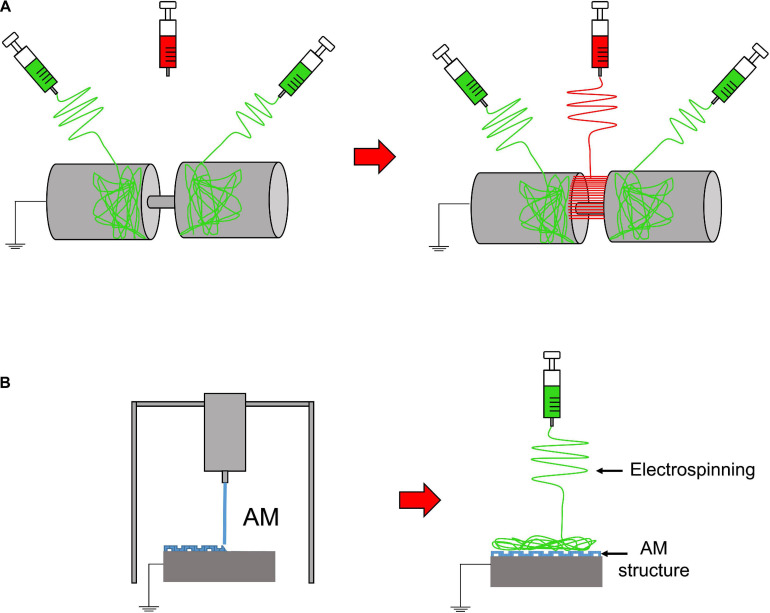
Electrospinning setups to realize 3D or composite structures. **(A)** Random-aligned setup: two drums with a gap in between them to obtain a random–aligned–random mat. **(B)** AM scaffold extruded on a flat plate collector, used to electrospun an additional mat on it.

Fine tuning all these parameters, it is possible to obtain several shapes, morphologies, and structures of the fibers, modifying also their mechanical properties (Ramakrishna et al., 2005). If these parameters do not reach an equilibrium, the resulting fibers can show the presence of beads on or just a spray of droplets can be produced (Ramakrishna et al., 2005, 2006; Haider et al., 2018). Other focal parameters to control the morphology and orientation of the nanofibers are the shape and movement of the ground collector (Figures 3, 4). The most common collector is the metallic flat plate. With such morphology, the nanofibers obtained have an isotropic random orientation (Figure 3A). The same arrangement can be obtained by using a drum collector rotating with a peripheral speed < 8 m s^–1^ (Figure 3B). The random mats such obtained were suitable to reproduce the structure of the cancellous bone or the fibrocartilage as well as their porosity (Jang et al., 2009; Bhattarai et al., 2018). When the peripheral speed is ≥ 8 m s^–1^ the nanofibers start progressively to be aligned along the circumference of the drum, obtaining a progressive anisotropic unidirectional orientation (Figure 3C). Another method to reach the same nanofiber orientation is the application of the “gap collector” setup (Figure 3D). The gap collector consists of two metallic rods or bars, placed at ground potential, separated by a free space. The nanofibers attracted by the ground potential of the two collectors will align, filling the gap. However, this method allows the alignment of the nanofibers only when a very limited gap is used (generally less than 100 mm). Moreover, after the removal of the aligned mat from the drum or gap collectors, it is possible to observe a shrinkage of the nanofibers. This property allows to confer to these uniaxial mats the typical morphology and nonlinear mechanical properties of the muscle and T/L tissues (Bosworth and Downes, 2011; Sensini and Cristofolini, 2018; Gotti et al., 2020a,b).

Furthermore, to develop gradients of random and aligned nanofibers or continuous regions with different materials, dedicated electrospinning procedures were designed. The multilayer electrospinning consists in the production of a first mat of nanofibers (random or aligned) and a further electrospinning of an additional mat on the previous one. For this process, the same or a different polymeric solution can be used (Figure 3E). The co-electrospinning consists in the simultaneous electrospinning of two different solutions on a drum or flat collector. This setup allows to obtain mats with two different types of nanofibers (Figure 3F). Both configurations were found to be suitable to mimic the fiber-orientation gradients of the enthesis and the MTJ (Ladd et al., 2011; Xie et al., 2012; He et al., 2017). Moreover, some groups modified these setups to obtain defined regions with completely different materials suitable to mimic the T/L–bone interface (Figure 4A) (Samavedi et al., 2014). In this case, scaffolds with a central aligned region and two random sides were obtained by electrospinning different solutions on two connected drums, rotating at low speed (< 8 m/s) (random nanofibers = bony sides). The central gap between the drums was suitable to produce aligned nanofibers, resembling the T/L tissue arrangement (Samavedi et al., 2014). Generally, electrospinning produces mats of nanofibers but, changing the ground collector setup, it is possible to obtain wires of axially aligned (i.e., bundles) or twisted (i.e., yarns) nanofibers (Abbasipour and Khajavi, 2013). In particular, bundles have an important role in the reproduction of fibers and fascicles of muscles and T/L (O’Connor and McGuinness, 2016; Sensini and Cristofolini, 2018; Sensini et al., 2019a,b). Moreover, some researchers combined electrospinning with other additive manufacturing (AM) technologies to better reproduce the structure of the biological tissues (Figure 4B). In fact, AM cannot produce nanometric fibers, while electrospinning has relevant limitations in mimicking the mechanical behavior of the bone tissue (Dalton et al., 2013). The materials used in electrospinning processes for tissue engineering purposes are several (Table 2). Researchers have also matched blends or core/shell of biomaterials to enhance the biocompatibility and the mechanical properties of the nanofibers (Table 3). To increase the bioactivity and speed up the cellular proliferation, the use of the nanoparticles, drugs and growth factors found a widespread employment (Table 4).

**TABLE 2:** Bulk materials used to electrospin scaffolds for the enthesis and MTJ regeneration.

Acronym	Extended name	Applications	References
PLGA	Poly(lactic-co-glycolic acid)	Tendon–bone	Li et al., 2009; Xie et al., 2010; Liu et al., 2011; Kolluru et al., 2013; Lipner et al., 2014, 2015; Zhao et al., 2014; Su et al., 2019
		Ligament–bone	Samavedi et al., 2014; Criscenti et al., 2016; He et al., 2017; Jiang et al., 2020
PLGA	Poly(l-lactide-co-glycolic acid)	Tendon–bone	He et al., 2014
PLGA	Poly(D,L-lactide-co-glycolic acid)	Tendon–bone	Spalazzi et al., 2008; Inui et al., 2012; Chou et al., 2016
PLLA	Poly(L-lactic acid)	Tendon–bone	Zhao et al., 2015; Baudequin et al., 2017; Li et al., 2017; Perikamana et al., 2018
		Tendon–muscle	Ladd et al., 2011
PCL	Poly(ε-caprolactone)	Tendon–bone	Li et al., 2009; Xie et al., 2012; Han et al., 2015, 2019; Bayrak et al., 2016; Baudequin et al., 2017; Nowlin et al., 2018; Wu et al., 2018; Zhu et al., 2019; Lin et al., 2019; Song et al., 2019; Reifenrath et al., 2020
		Ligament–bone	Samavedi et al., 2011, 2012, 2014; Criscenti et al., 2016; Lin et al., 2017; Olvera et al., 2017, 2020
SF	Silk fibroin	Tendon–bone	Zhi et al., 2016; Cai et al., 2018
PD	Polydopamine	Tendon–bone	Perikamana et al., 2018; Lin et al., 2019
PEUU	Poly(ester urethane urea)	Tendon–bone	Huang et al., 2020
PUR	Polyurethane	Ligament–bone	Samavedi et al., 2012
BPUR	Biodegradable poly(ether ester urethane urea)	Tendon–bone	Kishan et al., 2017
PEUUR2000	Poly(ester urethane urea) elastomer	Ligament–bone	Samavedi et al., 2011
Gelatin	Gelatin	Tendon–bone	Li et al., 2009; Zhao et al., 2015
CS	Chitosan	Tendon–bone	Wu et al., 2018; Han et al., 2019; Reifenrath et al., 2020
HA	Hyaluronic acid	Tendon–bone	Han et al., 2019
Col	Collagen	Tendon–bone	Han et al., 2015; Chou et al., 2016; Lin et al., 2019

**TABLE 3:** Blends and core–shell electrospun materials and respective fields of application.

Acronym	Type	Applications	References
SF/P(LLA-CL)	Blend	Tendon–bone	Cai et al., 2018
PCL/CS	Blend	Tendon–bone	Wu et al., 2018
PCL/Col	Blend	Tendon–bone	Han et al., 2015; Lin et al., 2019
		Tendon–muscle	Ladd et al., 2011
PCL/PLLA	Core–shell	Tendon–bone	Baudequin et al., 2017
CS/HA	Blend	Tendon–bone	Han et al., 2019
PLGA/Col	Blend	Tendon–bone	Chou et al., 2016
Li+@MSNs/PEUU	Blend	Tendon–bone	Huang et al., 2020
PLLA/Col	Blend	Tendon–muscle	Ladd et al., 2011

**TABLE 4:** Drugs and particles used to functionalize electrospun scaffolds for the enthesis and MTJ regeneration.

Acronym	Extended name	Applications	References
bFGF	Basic fibroblast growth factor	Tendon–bone	Zhao et al., 2014
BMP-2	Bone morphogenetic protein 2	Tendon–bone	Lipner et al., 2015; Han et al., 2019
		Ligament–bone	He et al., 2017; Jiang et al., 2020; Olvera et al., 2020
PDGF-BB	Platelet-derived growth factor-BB	Tendon–bone	Perikamana et al., 2018
SDF-1α	Stromal cell-derived factor 1	Tendon–bone	Han et al., 2019
TGF-β3	Transforming growth factor beta-3	Tendon–bone	Reifenrath et al., 2020
		Ligament–bone	Jiang et al., 2020
GO	Graphene oxide	Tendon–bone	Su et al., 2019
KGN	Kartogenin	Tendon–bone	Zhu et al., 2019
Li+	Lithium	Tendon–bone	Huang et al., 2020
Melatonin	Melatonin	Tendon–bone	Song et al., 2019
nHap	Hydroxyapatite	Tendon–bone	Liu et al., 2011; Kolluru et al., 2013; He et al., 2014; Han et al., 2015; Bayrak et al., 2016; Li et al., 2017; Wu et al., 2018
		Ligament–bone	Samavedi et al., 2011, 2012; He et al., 2017; Jiang et al., 2020; Olvera et al., 2020

## Results of the Literature Search

### Overview of the Electrospun Scaffolds for the Enthesis and MTJ Regeneration

In this section, a critical analysis of the state of the art of the most relevant interfacial tissue engineering works is reported. Each paper is classified according the hierarchical complexity of the scaffolds presented: simple mats (section “Simple Mats”); biphasic mats (section “Biphasic Mats”); multilayer mats (section “Multilayer Mats”); and composites and 3D structures (section “Composite and 3D Structures”). At the end of the present section, three tables are reported summarizing the results in terms of *in vitro* cell cultures (Table 5), *in vivo* experiments (Table 6), and mechanical properties (Supplementary Table 1) of the scaffolds described in each work.

**TABLE 5:** *In vitro* cell cultures developed on electrospun scaffolds for the enthesis and MTJ regeneration.

Cell type	Time point (days)	Culture type	Applications	References
Rat tendon fibroblasts	3–7	Static	Tendon–bone	Xie et al., 2010
Myoblasts (C2C12)	3–7	Static	Muscle–tendon	Ladd et al., 2011
Fibroblasts (NIH3T3)	3–7	Static	Muscle–tendon	Ladd et al., 2011
Murine calvarial preosteoblasts (MC3T3-E1)	3	Static	Tendon–bone	Li et al., 2009
	7	Static	Ligament–bone	Samavedi et al., 2011
	1–3–4–5–7–10	Static	Tendon–bone	Han et al., 2015
	2–6–10	Static	Ligament–bone	He et al., 2017
	1–3–5	Static	Tendon–bone	Huang et al., 2020
Rat bone marrow mesenchymal stem cells (rBMMSC)	1–4–7	Static	Tendon–bone	Zhu et al., 2019
Rat bone marrow-derived stromal cells (rBMSCs)	1–7–14–21–28	Static	Ligament–bone	Samavedi et al., 2012
	1–3–5–7–14–30	Static	Tendon–bone	Han et al., 2019
	1–4–7–14–28	Static	Ligament–bone	Jiang et al., 2020
	3	Static	Ligament–bone	Samavedi et al., 2014
Murine embryo fibroblast (C3H10T1/2)	1–3–7	Static	Tendon–bone	Zhao et al., 2015
	4	Static	Tendon–bone	Baudequin et al., 2017
Human dermal fibroblasts (HDFs)	1–3–5	Static	Tendon–bone	Zhao et al., 2014
Human adipose-derived stem cells (hADSCs)	3–7	Static	Tendon–bone	Xie et al., 2012
	1–3–5–7	Static	Tendon–bone	Perikamana et al., 2018
Human bone marrow mesenchymal stem cells (hBMMSCs)	3	Static	Tendon–bone	Kishan et al., 2017
	1–3–5–7	Static	Ligament–bone	Criscenti et al., 2016
	0–1–3–5–21	Static	Tendon–bone	Song et al., 2019
	1–7–14–21	Static	Ligament–bone	Lin et al., 2017
Rabbit bone marrow mesenchymal stem cells	1–3–5–7	Static	Tendon–bone	Zhi et al., 2016
	1–3–7–14	Static	Tendon–bone	Su et al., 2019
Bone marrow porcine mesenchymal stem cells (pBMMSCs)	0–10–20–21	Static	Ligament–bone	Olvera et al., 2017
	0–1–10	Static	Ligament–to–bone	Olvera et al., 2020
Osteosarcoma (not specified)	1–4	Static	Tendon–to–bone	Nowlin et al., 2018
Fibroblasts (not specified)	1–4	Static	Tendon–to–bone	Nowlin et al., 2018
Human osteoblast cells (HOS)	2	Static	Tendon–to–bone	Wu et al., 2018
Murine embryonic fibroblasts (3T3-L1)	1–3–5	Static	Tendon–to–bone	Huang et al., 2020
Rat tendon stem/progenitor cells (TSPCs)	1–3–4–5–7–14	Static	Tendon–to–bone	Lin et al., 2019

**TABLE 6:** *In vivo* tests on electrospun scaffolds for the enthesis and MTJ regeneration.

Animal	Time point (weeks)	Surgical site	Applications	References
**Rats**				
144	2–4–8	RCT	Tendon–bone	Zhao et al., 2015
144	2–4–8	RCT	Tendon–bone	Zhao et al., 2014
144	2–4–8	RCT	Tendon–bone	Huang et al., 2020
135	2–4–8	RCT	Tendon–bone	Zhu et al., 2019
93	2–4–8	RCT	Tendon–bone	Song et al., 2019
17	8	RCT	Tendon–bone	Reifenrath et al., 2020
64	2–4–8	RCT	Tendon–bone	Lipner et al., 2015
**Rabbits**				
24	4–8	ACL	Ligament–bone	Han et al., 2015
42	0–4–8–16	RCT	Tendon–bone	Inui et al., 2012
32	6–12	ACL	Ligament–bone	Zhi et al., 2016
48	8–16	Long digital extensor tendon	Tendon–bone	Chou et al., 2016
10	12	ACL	Ligament–bone	He et al., 2017
144	4–8–12	RCT	Tendon–bone	Li et al., 2017
90	6–12	Achilles tendon	Tendon–bone	Cai et al., 2018
48	4–8	ACL	Ligament–bone	Han et al., 2019
108	4–8–12	RCT	Tendon–bone	Su et al., 2019

### Simple Mats

**FIGURE 5:**
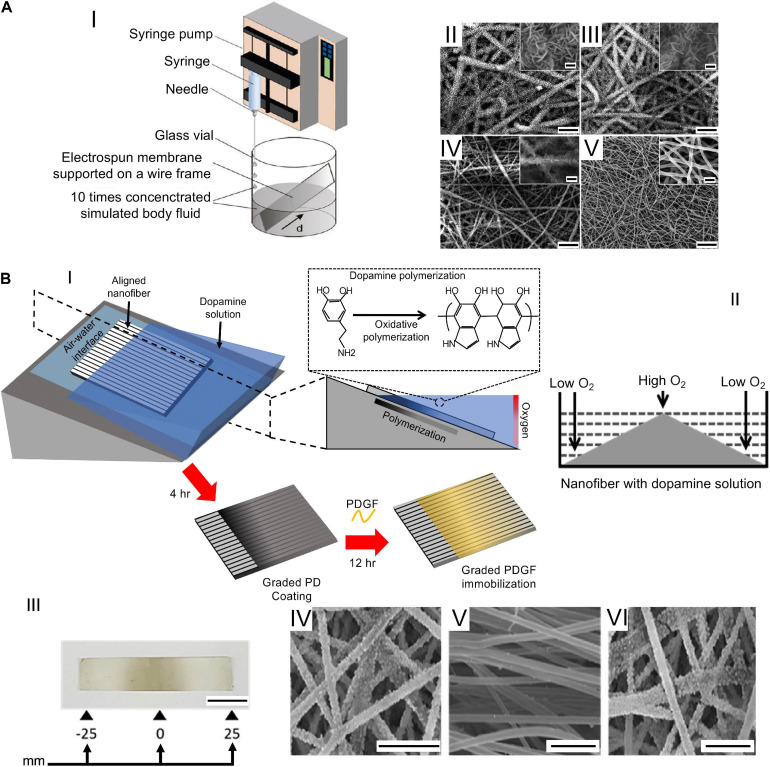
Examples of graded mats. **(A)** The setup and SEM images adapted from Li et al. (2009) (reproduced with permission. Copyright 2009, American Chemical Society): (AI) the setup used (d = scaffold distance from the bottom edge of the substrate); (AII-AV) SEM images taken at different d values from 0 (AII), 6 (AIII), 9 (AIV), and 11 mm (AV) (scale bar = 20 μm, insets scale bar = 2 μm). **(B)** Fabrication of a nanofibrous scaffold to mimic the bone–patellar tendon–bone structure: (BI) Production of the PD gradient re-drawn from Perikamana et al. (2018); (BII) symmetrical PD gradient generation on PLLA nanofiber surface; (BIII) image of the symmetrical gradient obtained (scale bar = 10 mm); (BIV–BVI) SEM images of the graft displaying mineralization at the ends and no minerals in the centre (scale bar = 10 μm) adapted from Perikamana et al. (2018) (reproduced with permission. Copyright 2018, Elsevier Ltd.).

Applying a bottom-up approach, some groups started to mimic the fibrous arrangement of the tissue interfaces, by electrospinning 2D nanofibrous mats. In a preliminary study Li et al. (2009) to mimic the tendon–bone junction, fabricated random nanofibrous mats coated with a continuous gradient of calcium phosphate. The scaffolds were produced by electrospinning PCL or PLGA random nanofibers on a flat plate collector. The membranes were plasma-treated and then mineralized with a ten-times simulated body fluid (10SBF) from 2 to 6 h (Figure 5A), creating a mineral gradient. The mechanical tests were carried out using a digital image correlation approach and evaluated only on stripes of PLGA mats. After the application of different stress values, the PLGA specimens showed an increment of strain when the mineral content was reduced. Conversely, the stiffness decreased with the decrease in the mineral content. For the *in vitro* culture, only the PCL random mats with the mineral gradient were seeded with mouse calvarial-derived preosteoblastic cells (MC3T3-E1). Before the cultures, the PCL mats were also covered with gelatin. The cellular proliferation analysis showed, after 3 days of culture, a heightened cell density on the mineralized side (Li et al., 2009). With the same purpose, Liu et al. (2011) applied a new method to mineralize an electrospun mat of PLGA. They produced aligned nanofiber mats by electrospinning a PLGA solution on a rotating drum collector. Before the mineralization procedure, they were bathed in a watery solution of chitosan and 1-ethyldimethylaminopropyl carbodiimide (EDC), N-hydroxysuccinimide (NHS), 2-morpholinoethane sulfonic acid (MES). Then they were immersed in a 1% heparin mixed with a solution of EDC-NHS-MES. The mats were finally immersed into a modified 10-times concentrated simulated body fluid (m10SBF), for different time intervals (i.e., to cover them with Hap). They found that the stiffness increased with the mineral content, while no statistical differences were observed for the yield stress. This new method of mineralization showed a denser and thicker coating than the previous works, even if the mechanical performances were lower than the bone tissue (Liu et al., 2011). In another study, Inui et al. (2012) studied the effects of the enthesis healing in an infraspinatus rabbit model. They fabricated random mats of PLGA nanofibers that were implanted in 42 rabbits. After 16 weeks, the histological tests revealed an expression of collagen Types I, II, and III (tendon side) and new cartilage formation (enthesis side). The biomechanical tests on an infraspinatus tendon–scaffold–humeral head complex showed no statistically significant differences in the stiffness and load to failure between the group treated with the scaffolds and the control one (Inui et al., 2012). Kolluru et al. studied the mechanical behavior of as-spun, mineralized, and unmineralized nanofibers designed for the healing of tendon–bone enthesis. PLGA nanofibers were collected on metallic frames placed close to a flat aluminum collector. The unmineralized nanofibers were treated with a plasma cleaner, obtaining three different shapes: (i) a uniform circular cross section with sparse surface irregularities; (ii) a uniform ellipsoidal cross section; and (iii) non-uniform/rough cross section along the entire nanofiber length. The mineralized ones were obtained by soaking the nanofibers at different time points in 10SBF. This procedure allowed to obtain three increasing thickness of mineralized Hap coatings, called respectively: (i) thick-platelet mineral coating; (ii) thick-conformal mineral coating; and (iii) thin-conformal mineral coating. The micromechanical tests, carried out on the single fibers, showed that the tensile strength was higher for the unmineralized groups as well as the yield stress. No statistical difference was found instead, in the stiffness (Kolluru et al., 2013). Zhao et al. (2014) concentrated their studies on the rotator cuff tear (RCT), fabricating a random PLGA electrospun membrane, loading the nanofibers with a basic fibroblast growth factor (bFGF). The *in vitro* cell culture was carried out with human dermal fibroblasts (HDFs). After 5 days of culture, the scaffolds revealed a higher cellular proliferation in the bFGF-PLGA membranes compared to the pure PLGA ones. The *in vivo* study was carried out on three groups of rats (control group, PLGA group, and bFGF-PLGA group). After 8 weeks of implantation, the histological analysis revealed the formation of fibrocartilaginous tissue and a significantly greater area of glycosaminoglycan (GAG). Moreover, a higher collagen organization for the bFGF-PLGA group was noted. The biomechanical tests pointed out the increase of mechanical properties with time in the bFGF-PLGA samples, after 8 weeks of implantation (Zhao et al., 2014). Later, Zhao et al. (2015) continued their work on RCT, fabricating electrospun random PLLA membranes grafted with gelatin. They lyophilized and sterilized them before the gelatin modification, which was achieved with an aminolysis method. The cell cultures (murine embryo fibroblast: C3H10T1/2) showed a higher proliferation on the gelatin–PLLA compared to the pure PLLA group. In a large *in vivo* test, performed on a RCT rat model, the animals were divided in three groups (control group, PLLA group, and gelatin–PLLA group). The histological analysis showed that on the gelatin–PLLA membranes, after 8 weeks of implantation, a greater GAG staining area and a boost in the new cartilage formation were noted. The biomechanical tests confirmed the promising outcomes of the gelatin–PLLA mats after 8 weeks of trials: a higher failure load, stiffness, and failure stress compared to the other groups (Zhao et al., 2015). Lipner et al. (2014) focused on aligned PLGA electrospun scaffolds with a gradation in mineral content for the tendon–bone insertion. The scaffolds were electrospun on a rotating drum collector, cut into pieces, and plasma treated (increasing their hydrophilicity). Subsequently, two different groups were produced: a first one was mineralized in a modified 10SBF and then incubated with heparin, chitosan, and the EDC cross-linker; the second one was simply mineralized with 10SBF. The mineralization with 10SBF appeared plate-like and diffuse, while the modified 10SBF mineralization was dense and conformal to the fibers (Lipner et al., 2014). The mechanical tests revealed a higher strain on the lower mineralized region. The denser mineral coating of the m10SBF samples resulted in an increased stiffness compared to the 10SBF group (Lipner et al., 2014). In a later work, Lipner et al. (2015) continued their previous study by seeding the same PLGA mats with adipose-derived stem cells (ADSCs), before the implantation in a rat model. After the electrospinning process, the mats were plasma treated, mineralized with 10SBF, and implanted on 64 rats. One group of scaffolds was realized by loading the nanofibers with nanoparticles of bone morphogenic protein 2 (BMP2). The scaffolds were divided in four groups: control, acellular, cellular, and cellular-BMP2. The histologic analysis revealed, after 8 weeks, a delayed healing response for all the scaffold groups, compared to the suture-only control group. Moreover, the mechanical properties, after 8 weeks of *in vivo* test, were significantly decreased for all the scaffolds compared to the control. In general, the scaffolds showed lower biological and mechanical performances in the enthesis regeneration compared to the control group, suggesting that BMP2 is not a good candidate for the enthesis healing (Lipner et al., 2015). Han et al. (2015) produced a random PCL/nHAP/Col membrane to promote tendon–bone healing. The blend was electrospun on a flat aluminum collector. The mats were cut in circular specimens and seeded with MC3T3-E1 cells. A preliminary *in vivo* study on rabbits ACL was performed on 2 groups (PCL/nHAP/Col and pure PCL). The histological analysis demonstrated a higher proliferation rate for PCL/nHAP/Col membrane and new bone formation after 8 weeks. Moreover, the mechanical properties of the PCL/nHAP/Col group were higher than the pure PCL ones. This study combined three biomaterials for the tendon–bone healing: the PCL as bulk material; the nHap to speed up the bone regeneration; and the Col to enhance the T/L healing (Han et al., 2015). Bayrak et al. (2016) used a multiple-spinneret electrospinning to fabricate mats to promote the tendon–bone repair. A solution of PCL and a suspension of nHap were loaded in opposite syringes and co-electrospun on a rotating drum collector. This allowed to obtain random mats of PCL nanofibers with nHap particles in between. Fiber diameters and pore size were analyzed showing an increased fiber diameter for nHap-loaded nanofibers, but no significant differences on the mean pore size. The contact angle was smaller in the nHap-loaded nanofibers, suggesting the hydrophilicity of nHap (Bayrak et al., 2016). In another study, Zhi et al. (2016) tested the influence of electrospun random mats of silk fibroin (SF) to enhance the tendon–bone healing in a rabbit model. The mats were cut in strips and seeded with rabbit bone marrow mesenchymal stem cells. The *in vitro* test indicated a better proliferation on the nanofibrous membranes compared to simple mats. In the *in vivo* study, rabbits’ Achilles tendons were wrapped with an SF mat and compared with a control untreated group. The histological analysis revealed new bone formation in the SF mat group and a complete absorption of mats after 6 weeks. The biomechanical properties after 12 weeks resulted higher, in terms of failure load, in the SF group compared to the control one (Zhi et al., 2016). Studying suitable biomaterials for the interfacial tissue engineering, Kishan et al. (2017) produced gradient mats of biodegradable polyester urethane urea (BPUR) at different weight ratios (BPUR50: 50% wt; BPUR10: 10% wt). The solution of BPUR10 had a decreasing flow rate, the BPUR50 an increasing one. This method allows to produce scaffolds with a gradient of nanofibers along the mats thickness (Kishan et al., 2017). To fabricate random gradient mats, an aluminum wheel was used rotating them of 90° over 6 h. For the aligned gradient mats instead, a collector wheel with 8 parallel copper wires was used. After a 3-day culture, the human bone marrow mesenchymal stem cells (hBMMSCs) were aligned along the fibers’ direction on the aligned meshes while no particular orientation was observed in the random ones. BPUR10 had the lowest stiffness and the highest failure strain, with respect to the BPUR50 region. This study introduced the concept of materials gradient along the thickness of the mat, a focal point for a scaffold for the enthesis regeneration (Kishan et al., 2017). Chou et al. combined a biodegradable collagen-loaded random membrane and a 3D-printed anchoring bolt to promote tendon–bone repair. The nanofibrous mat was produced by electrospinning a blend of PLGA/Col at different (v/v) ratios (40/60 v/v, 50/50 v/v, 67/33 v/v, 80/20 v/v, 100/0 v/v). The human fibroblasts had a higher proliferation rate on the 67/33 v/v mats and, for this reason, were chosen for the *in vivo* test. The random mats were sutured on the side of the long digital extensor tendon inside a dedicated bone tunnel and compared with a control group. The histological analysis showed new bone formation and the biomechanical tests a higher failure load for the bolt+mat group compared to the control after 16 weeks (Chou et al., 2016). Baudequin et al. (2017) analyzed the ability of different PCL and PLLA mats (pure or core–shell) to induce the tendon–bone cell differentiation. With this aim, PCL and PLLA were separately electrospun to obtain aligned and random mats of nanofibers (i.e., random PCL, aligned PCL, random PLLA) and also random core–shell mats of PCL/PLLA (PCL out-PLLA in; PLLA out-PCL in). In the mechanical tests, the core–shell scaffolds scored the highest stiffness. C3H10T1/2 fibroblasts were used for the *in vitro* cultures. The cellular proliferation on the PCL scaffold was the most continuous and dense. Bone [distal-less (Dlx5), runt-related transcription factor 2 (Runx2), bone gamma-carboxyglutamate protein (Bglap)], and tendon [scleraxis (Scx), tenomodulin (Tnmd), aquaporin 1 (Aqp1)]-related marker expression was also analyzed. PCL membranes had the highest values of Bglap and the lowest values of Scx. The core–shell mats improved the tenogenic differentiation with a significant Tnmd value. The PLLA scaffolds were found less suitable to induce tendon or bone differentiation, since a decrease in the Scx, Tnmd, and bone markers was observed (Baudequin et al., 2017). Olvera et al. (2017) focusing on the ligament–bone healing examined the effect of the growth factor stimulation on mesenchymal stem cells. The bone marrow porcine mesenchymal stem cells (pBMMSCs) were seeded on random and aligned microfibrillar mats of PCL, obtained by electrospinning onto a rotating drum collector. The scaffolds were individually or sequentially stimulated with transforming growth factor (TGF-β3) (chondrogenic medium) and connective tissue growth factor (CTGF) (ligamentum medium). Ligamentous (Col1a1, Col3a1, actin alpha 2 (acta2) and Tnmd) and chondrogenic (Col2a1, Col10a1, aggrecan (ACAN), and SRY-Box 9) markers were evaluated. The scaffolds stimulated with TGF-β3 showed higher collagen Type II expression on the aligned fibers compared to randomly oriented ones. The bone morphogenic protein (BMP-2) and collagen Type I expression was higher in the random mats. A ligamentous differentiation was observed on the CTGF stimulated aligned scaffolds with a higher expression of Tnmd (Olvera et al., 2017). Wu et al. realized a nanofibrous mesh for T/L to bone interface and explored their biological properties. Random nanofiber mats were fabricated by electrospinning a blend of PCL/CS loaded with nHap on a rotating drum collector. Pure PCL/CS scaffolds were also produced as a control. The nHap-loaded scaffolds showed greater adhesion and proliferation of human osteoblasts (HOS) after 2 days of culture. They had higher mechanical properties compared with the pure PCL/CS ones. In particular, the stiffness of the nHap-loaded mesh was similar to that of the ligament tissue (Wu et al., 2018). Perikamana et al. continued to investigate the tendon–bone repair, producing dedicated PLLA nanofibrous mats. The mats were fabricated, electrospun with a PLLA solution on a metal collector rotating at different rates, to obtain random and aligned fibers. The nanofibers were coated with polydopamine (PD) and with platelet-derived growth factors BB (PDGF-BB). A total of 4 groups of scaffolds were produced: PD-coated (random or aligned) mats and PDGF immobilized PD-coated (random or aligned) mats. After 3 days of culture, human adipose-derived stem cells (hADSCs) revealed a higher proliferation in the PDGF groups. The DNA assay was carried out at days 1, 3, 5, and 7, showing higher and increasing values of Scx, Tnmd, and decorin expression for the PDGF-immobilized groups. Moreover, the Rho/Rock pathway aspect was also analyzed, by blocking the Rock1 signal with a pharmacological inhibitor (Y-27632). A drastic disruption of the cytoskeleton structure and a reduction of the tenogenic gene expression were observed in all groups. Moreover, they found that if Rock1 was blocked, the contribution of the nanofiber alignment on the cell differentiation was negligible. After these results, a bone–patellar tendon–bone graft was produced. They produced a random–aligned–random scaffold that was immersed in a PD solution. To obtain a gradient of PD, the scaffold was immersed in a folded “V” shape. This allowed to have the center part with the highest amount of PD coating, while the edges were less coated (Figures 5B II–III). Moreover, using a syringe pump infusion method the edges of the scaffold were mineralized (bone side). This scaffold was also able to induce osteogenic differentiation, showing a significantly higher expression of alkaline phosphatase (ALP) (Perikamana et al., 2018). Huang et al. (2020) directed their study in enhancing tendon–bone healing, by developing a random nanofibrous membrane. In particular, they investigated the topic of fat infiltrations. The mats were realized by electrospinning a blend of PEUU and a solution of mesoporous silica nanoparticles doped with lithium (Li+ @ MSNs). PEUU and Li+/PEUU scaffolds were produced to compare them with the Li+@MSNs/PEUU group. MC3T3-E1 and 3T3-L1 (murine embryonic fibroblasts) cell lines were seeded and cultured on the scaffolds up to 5 days. The cytocompatibility assay showed a significant difference between the PEUU group and the control group with very few dead cells which were observed after 5 days. This was confirmed in the Li+ concentration analysis, where it was found that low concentrations of Li+ could enhance the proliferation and osteogenic differentiation and also inhibit the audiogenic differentiation. A high concentration of Li+ instead inhibited the proliferation of cells. For the rat *in vivo* study, the samples were divided into four groups (suture only, PEUU mat, Li+/PEUU mat, Li+@MSN/PEUU mat) and investigated up to 8 weeks. The mechanical tests on scaffolds showed that the Li+@MSN/PEUU group had the highest stiffness but also the lower tensile strength and strain at failure. The histological investigation documented the decreased fatty infiltration and an improvement of collagen organization in Li+ mats. The Li+@MSN/PEUU group after 8 weeks revealed the highest values of bone mineral density and bone production compared to the other categories. In addition, the biomechanical properties, which increased with time, evinced higher values for Li+ groups. Moreover, Li+@MSN/PEUU scored the highest values of load to failure, stiffness, and failure stress (Huang et al., 2020). Lin et al. (2019) to boost the tendon–bone healing, produced different random nanofibrous membranes, evaluating their behavior with rat tendon stem/progenitor cells (TSPCs). The membranes were divided into PCL, PCL/Col-I, PD-coated PCL, and PD-coated PCL/Col-1. The PCL/Col-1 membranes were obtained, electrospinning a blend of PCL and Col-I at different volume ratios (4:1 v/v, 2:1 v/v, 1:1 v/v, 1:2 v/v), while the coating was achieved by immersing the electrospun nanofibers in PD. The PD coating showed to promote the osteoblast adhesion and proliferation on these biodegradable polymers. The cytocompatibility assay revealed an increasing proliferation on the PCL/Col-I membranes (2:1) after 7 days of culture. The expression of osteogenic genes showed that PCL/Col-I (2:1) had the highest expression of Col1a1 marker at all time points and a significant increase of the osteocalcin (OCN) expression at 14 days. The Runx2 expression was significantly higher in the PCL/Col-I (4:1) and PCL/Col-I (2:1) groups. The PD-coated specimens did not promote further osteogenic marker expression compared to the pure PCL membrane (Lin et al., 2019). Song et al. (2019) focused on melatonin-loaded PCL membranes to speed up the tendon–bone repair. The aligned nanofibrous membranes were electrospun on a rotating drum, using a solution of PCL loaded with melatonin nanoparticles. Parallelly, pure PCL mats were fabricated as a control. A large animal trial was performed on rat RCT, dividing the animals into 3 groups (control, PCL, melatonin–PCL). The biological tests, carried out with hBMMSCs, proved the cytocompatibility of PCL membranes and a good melatonin release with a percentage of 77%. A larger collagen Type II area and a remarkable gene overexpression of ACAN and SRY-Box9 were observed in the melatonin–PCL mats. This provided that the melatonin release stimulated the chondrogenic differentiation. Moreover, new cartilage formation and a larger GAG area were observed, particularly in the melatonin–PCL group. The biomechanical tests showed increasing properties with time and that the melatonin–PCL samples scored the highest properties of failure load, stiffness, and failure stress after 8 weeks (Song et al., 2019). Zhu et al. (2019) studied the influence on the enthesis healing of a KGN-loaded PCL aligned nanofiber meshes. A group of 135 rats were used for the *in vivo* evaluation and divided into 3 categories (repair only, PCL membrane, KGN–PCL membrane). The KGN release analysis displayed a rate of 80% release by day 20. The biological tests carried out with rat bone marrow stromal cells (rBMMSCs) showed an increasing proliferation on all the PCL membranes. The study showed that an increasing amount of KGN could upregulate the chondrogenic differentiation. Conversely, no tenogenic differentiation improvement was observed in KGN-PCL groups compared to the pure PCL membranes. In the histological assay, new fibrocartilage was detected, which was more similar to the native enthesis in the KGN-PCL group. Moreover, the GAG investigation revealed that the KGN-PCL membranes had the highest GAG area at all time points. The biomechanical tests proved that the KGN-PCL mats had the highest failure load after 8 weeks (Zhu et al., 2019). Su et al. (2019) tried to improve the regeneration of the injured enthesis, fabricating a GO-doped PLGA random nanofiber mats. Random mats of PLGA/GO and pure PLGA were electrospun on an aluminum flat collector. The tensile tests on the scaffolds revealed that the tensile strength of PLGA was higher compared with the PLGA/GO. The *in vitro* tests showed an increased proliferation of rabbit bone marrow mesenchymal stem cells in the PLGA/GO compared to PLGA membranes. A similar trend was observed for the osteogenic differentiation, where more distinct mineralized nodules on the PLGA/GO membrane were observed. The scaffolds’ performances were tested in a *in vivo* RCT rabbit model divided in 3 groups (control, PLGA, PLGA/GO). Histomorphometric analysis displayed a larger new cartilage formation area in the PLGA/GO than the PLGA. The biomechanical properties of the supraspinatus tendon–humerus complex increased with time. The properties were significantly higher in the PLGA/GO compared with the other ones (Su et al., 2019). In another study, Reifenrath et al. (2020) reported the effect of a chitosan-coated PCL mat when loaded with TGF-β3. PCL-aligned mats were electrospun on a rotating drum collector. These scaffolds were modified with chitosan-grafted PCL (CS-graft-PCL), which was bounded to PCL using a self-induced crystallization method. Finally, the modified meshes were loaded with TGF-β3. The biomechanical tests performed on an *in vivo* RCT rat model revealed that the TGF-β3-loaded membranes had higher failure compared with the CS-g-PCL ones but were slightly lower than native control group values (Reifenrath et al., 2020).

### Biphasic Mats

**FIGURE 6:**
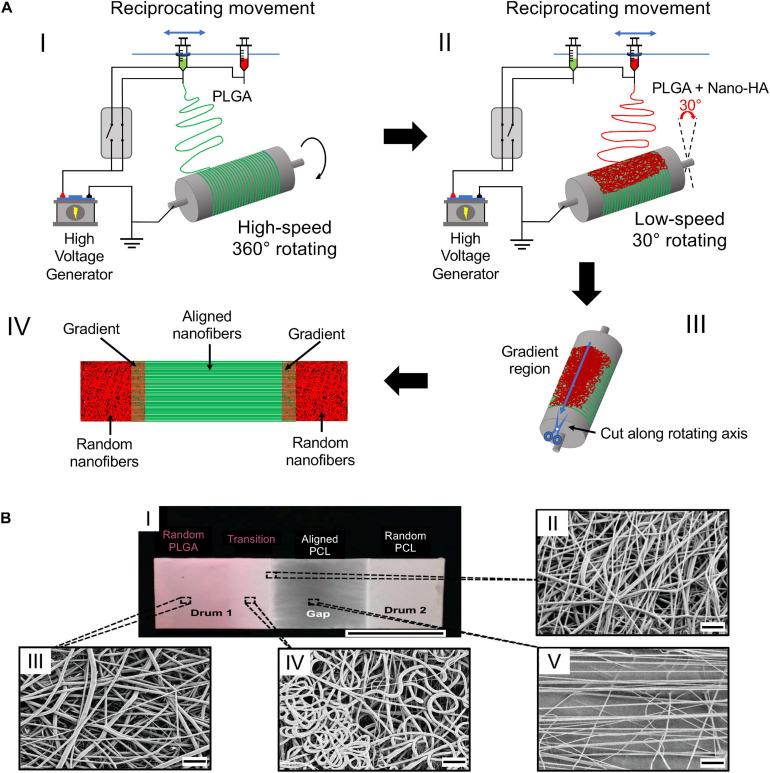
Electrospinning setup for the production of biphasic mats. **(A)** Electrospinning setup re-drawn from He et al. (2014): (AI) electrospinning of pure PLGA at high rotating speed to achieve the fiber alignment; (AII) electrospinning of nHap-PLGA at low speed to obtain random fibers; (AIII) mat cut along the axis; (AIV) final mat with the different regions. **(B)** Electrospun scaffold adapted from Samavedi et al. (2014) (reproduced with permission. Copyright 2014, Wiley Periodicals, Inc.): (BI) image of the electrospun scaffold; (BII–BV) SEM images of the mats: (BII) random PLGA region; (BIII) transition region; (BIV) aligned PCL region; (BV) edge of the transition region ((BI) scale bar = 2.5 cm, (BII–BV) scale bar = 10 μm).

To increase the biomimicry with the ECM of the natural interfaces, some groups have investigated scaffolds characterized by continuous linear zones with dedicated nanofiber arrangements. This biofabrication aspect is fundamental to expression of an anisotropic mechanical, morphological, and compositional behavior of the tissue junctions. To enhance the tendon–bone repair, Xie et al. (2010) proposed a method to electrospin a biomimetic aligned to random scaffold. The scaffold was created by electrospinning PLGA nanofibers on a gap collector, made of two stapler-shaped metal bars. This setup allowed to obtain aligned nanofibers on the gap (tendon site) and random ones on the metal bars (bone site). The cellular tests, performed with rat tendon fibroblasts, revealed that cells were axially aligned with the fibers (tendon site) while the ones on the random portion had a disorganized pattern (bone site). Moreover, collagen Type I was predominant compared to collagen Type II and oriented in the fiber direction (Xie et al., 2010). He et al. (2014) realized a scaffold with both structure and material gradients. Their aim was to reproduce the tendon–bone enthesis, matching random to aligned fibers and a gradation in mineralization (Figure 6A). The electrospinning configuration was based on the movement of two syringes above a rotating drum, which were alternately turned on. The first syringe contained pure PLGA, while the second contained nHap-PLGA. The drum rotated at different speeds to obtain random and aligned structures. The mats cut along the gradient direction, and analyzed via X-ray diffraction, confirmed a continuous gradation both in fiber organization and in the materials’ composition. Nowlin et al. (2018) realized a random to aligned mat to improve the tendon–bone enthesis regeneration. The PCL mats were electrospun on 2 parallel aluminum bars to obtain random (on the bar surface) and aligned (in the gap) nanofibers. The resulting scaffold was seeded with osteosarcoma cells (random region) and fibroblasts (aligned region), respectively. After 4 days of cellular growth, the fibroblasts resulted to be aligned and elongated along the nanofibers’ direction, while the osteosarcoma cells resulted to be randomly oriented. Moreover, a cell migration was observed forward the mixed region (Nowlin et al., 2018). Samavedi et al. (2011) focused on the ligament–bone regeneration. Random nanofibrous mats, with three different regions, were obtained by co-electrospinning on a drum collector a nHap-PCL and a PEUUR2000 solution. The newly produced mats were divided into three regions: nHap-PCL (bone), interface (enthesis), and PEUUR2000 (ligament). Then, some scaffolds were cut into pieces and mineralized with 5-times concentrated simulated body fluid (5SBF). The mechanical characterization showed a significant increase of the stiffness for the 5SBF-treated nHap-PCL samples compared with the as-spun ones. PEUUR2000 samples revealed a higher failure stress than nHap-PCL ones. The cell metabolic activity was evaluated with MC3T3-E1 cells revealing that PEUUR2000 had the highest absorbance in both cases (Samavedi et al., 2011). Samavedi et al. (2012) investigated the role of BMSCs and their impact on the ligament–bone interface, using a graded membrane. The scaffolds were fabricated by co-electrospinning of a nHap-loaded PCL solution and a PUR one on a rotating drum. The resulting scaffolds were cut into smaller pieces and divided into 3 regions (nHap-PCL, GRAD, PUR). The samples were treated with 5SBF to mineralize them. The *in vitro* tests with rat BMSCs (rBMSCs) revealed a higher metabolic activity in PUR samples, in particular in the unmineralized ones. The expressions of osteogenic markers BMP-2 and osteopontin (OPN) were higher in mineralized samples, whereas that of ALP was higher in unmineralized ones (Samavedi et al., 2012). Samavedi et al. (2014) deepened their previous study on the ligament–bone healing, designing a scaffold with regions differing for structure and composition Figure 6B). These scaffolds were realized by electrospinning PCL and PLGA at different (w/w) ratios: PCL (7.5%)–PLGA (13%) and PCL (10.5%)–PLGA (13%). The collector was composed of two drums connected by a metal rod. The PCL was electrospun in the gap between the drums obtaining aligned nanofibers. The PCL syringe was turned off, and PLGA electrospinning was started onto one drum. The mechanical tests showed that the PLGA random fibers had a significantly higher stiffness than the aligned PCL fibers. An opposite trend was observed for the failure stress which was approximately 2 times higher in PCL regions than PLGA ones. The rBMSCs’ morphological assay showed a randomic organization on the random nanofibers while they were aligned in the aligned region. The cells in the random region had a polygonal shape and an elongated one on the aligned fibers (Samavedi et al., 2014). Jiang et al. (2020) wanted to deeply study the differentiation capability of rBMSCs on the nanofibrous scaffolds. Mats of PLGA with a gradient of nHap-BMP2 were produced. The electrospinning setup was the same used by He et al. (2017) in their study. After electrospinning, TGF-β3 was uniformly added to the aligned nanofibers. The morphology, viability, and differentiation of cells was investigated after 7 days. The viability assay showed that cells had a uniform distribution. The morphological investigation highlighted a randomic ECM distribution on the random region since the first day after seeding. Concerning the differentiation study, the ALP was found to be promoted by nHap-BMP2 in the random nanofiber region. Moreover, higher levels of OCN and Runx2 expression were observed. An opposite trend was found for the Sox9 expression level, which was higher on the aligned region (Jiang et al., 2020).

Obviously, not every tissue junction has a mineralization gradient, as in the case of the MTJ. A preliminary study was made by Ladd et al. (2011) who used electrospinning to produce a scaffold to improve muscle–tendon junction repair. Random nanofibrous membranes were obtained, co-electrospinning a solution of PLLA/Col and a PCL/Col one on a drum collector. This allowed to obtain 3 regions (PCL side, PLLA side, overlap side). The mechanical tests showed that the scaffolds scored 7.3 ± 2.1 MPa in stiffness, 0.5 ± 0.2 MPa in failure stress, and 18.5 ± 8.2 % in strain at failure. These values were significantly higher compared to native MTJ, characterized by a stiffness of 0.3 ± 0.1 MPa, a failure stress of 0.1 ± 0.01 MPa, and a strain at failure of 122.4 ± 19.9%. The cell cultures, carried out with mouse myoblasts (C2C12) or mouse fibroblasts (NIH3T3), revealed the progressive formation of myotube and fibroblast proliferation (Ladd et al., 2011).

### Multilayer Mats

**FIGURE 7:**
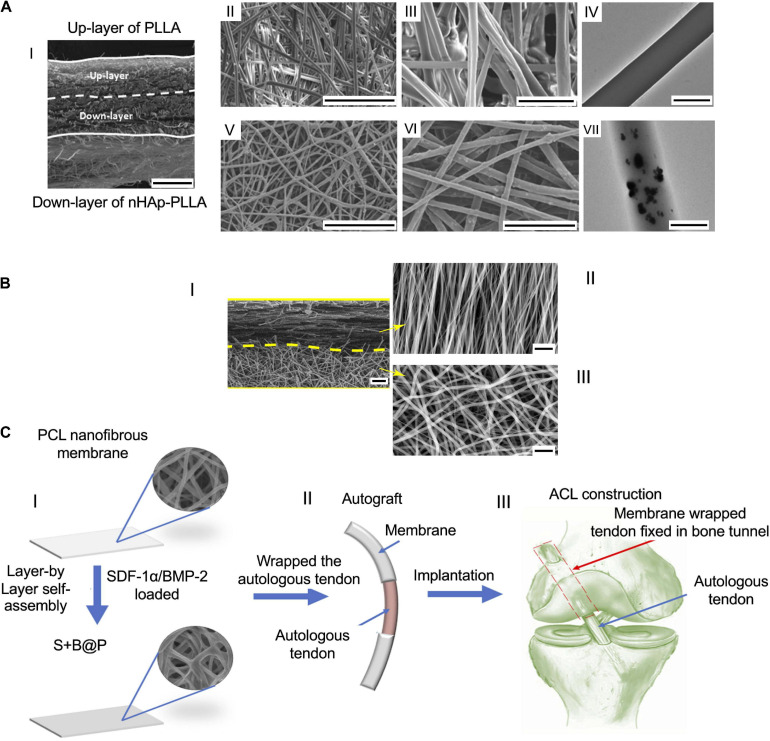
Examples of multilayer scaffolds. **(A)** SEM images of the dual layer flexible nanofibrous membrane adapted from Li et al. (2017) (reproduced with permission. Copyright 2017, Acta Materialia Inc.): (AI) cross section of the membrane (scale bar = 100 μm); (AII and AIII) upper layer of PLLA nanofibers ((AII) scale bar = 50 μm, (AIII) scale bar = 10 μm); (AIV) TEM image of a PLLA fibers (scale bar = 1 μm); (AV and AVI) down layer of nHap-PLLA fibers [(AV) scale bar = 50 μm; (AVI) scale bar = 10 μm]; (AVII) TEM image of a nHap-PLLA fiber (scale bar = 1 μm). **(B)** SEM images of the dual layer aligned-random scaffold adapted from Cai et al. (2018) (reproduced with permission. Copyright 2018, Dove Medical Press Limited): (BI) cross section of the mat (scale bar = 10 μm); (BII) aligned region (scale bar = 5 μm); (BIII) random region (scale bar = 5 μm). **(C)** Images of a layer-by-layer assembly adapted from Han et al. (2019) (reproduced with permission. Copyright 2019, Dove Medical Press Limited): (CI) Fabrication of the nanofibrous mat; (CII) mats wrapped up on the autologous tendon ends; (CIII) implantation of the graft.

A focal aspect of the tissue junctions is to guarantee a 3D volumetric distribution of the tissue gradient. For this reason, the multilayer configuration, conversely to the biphasic mats, is thought to mimic the natural volumetric gradient of nanofiber organizations and materials. In a preliminary study, Xie et al. produced a scaffold with structural gradients to mimic the tendon–bone insertion site. They electrospun aligned PCL nanofibers on a gap collector. Then, the aligned mats were transferred to a glass coverslip serving as a collector for electrospinning of random PCL fibers. The scaffolds thus obtained were seeded with hADSCs and analyzed up to 7 days. The cell morphology showed a homogeneous cell organization on the random region, while they resulted to be aligned along the aligned nanofibers (Xie et al., 2012). Due to improvement of the tendon–bone repair, a dual-layer random electrospun mat, with a mineralization gradient, was designed by Li et al. (2017) (Figure 7A). The scaffold was prepared, electrospinning PLLA and nHap-PLLA nanofibers on a rotating drum collector. A mat of PLLA-nHap was spun on a first layer of pure PLLA, obtaining a dual-layer structure. To mineralize the membranes thus obtained, they cut them into pieces immersing them in an SBF solution. An *in vivo* study was performed on 144 rabbits which were divided into 3 groups: control, PLLA simple nanofibrous membrane (SFM), and bipolar fibrous membrane (BFM). The histological analysis revealed a greater GAG staining area in the scaffold groups compared with the control one. Moreover, the ability of BFM to improve cartilage regeneration and the collagen organization after 12 weeks was found. New bone formation and higher tendon maturing score (TMS) were observed in particular in the BFM after 12 weeks. The biomechanical tests displayed increasing properties with time. In fact, at 12 weeks the failure load was significantly higher in the BFM group than in the other ones. A similar trend was observed with the failure stress values, which were found greater in the BFM group. Cai et al. focused on the tendon–bone enthesis, investigating the effect of an aligned-random mat (ARM) of SF/P(LLA-CL) on the enthesis healing (Figure 7B). They fabricated a random mat on a drum collector, and then the rotational speed was increased to obtain an overlapped aligned layer. Random nanofibers mats were also prepared as control (RM). The resulting scaffolds were cross-linked with alcohol to remove the residual solvent. An *in vivo* rabbit Achilles trial was carried out dividing the animals in 3 groups: control (unwrapped tendon transplantation) and experimental (tendon wrapped with RM or ARM). The histological assay showed oriented collagen Type I fibers and a larger area of GAG in the ARM group, while new bone formation was revealed in both scaffold groups. Collagen Type III was significantly higher in the RM group and in the control one. The biomechanical tests revealed that the mechanical behavior increased over time. However, the properties were higher for the experimental groups at every time point. In particular, at 12 weeks the ARM showed the highest values of failure load and stiffness (Cai et al., 2018). A multilayer random nanofibrous scaffold was designed by Han et al. (2019) with the aim to improve the tendon enthesis regeneration after a surgical autograft implantation. Random PCL membranes were electrospun on a drum collector. The scaffolds thus obtained were coated with a CS/HA film, loaded with stromal cell-derived factor 1-α (SDF-1α) and BMP-2 (Figure 7C). The coating was achieved by a layer-by-layer self-assembly method, soaking firstly the PCL membrane in a SDF-1α and HA solution, and then in a CS and a recombinant human BMP-2 (rhBMP-2) one. This membrane was denoted as S+B@P. BMP-2-loaded PCL random mats (B@P) were also fabricated. The *in vitro* cell culture was carried out with rBMSCs. The cell viability and proliferation showed a higher growth and migration for the S+B@P group. Similar trends were found for the osteogenic differentiation analysis with a higher gene expression of Runx2, OCN, Col-I, and OPN on the S+B@P group. A rat ACL model was performed dividing the animals in 3 groups (PCL, B@P, and S+B@P). In the biomechanical tests, the S+@P group showed improved mechanical properties compared with the B@P and PCL groups after 12 weeks (Han et al., 2019).

### Composite and 3D Structures

**FIGURE 8:**
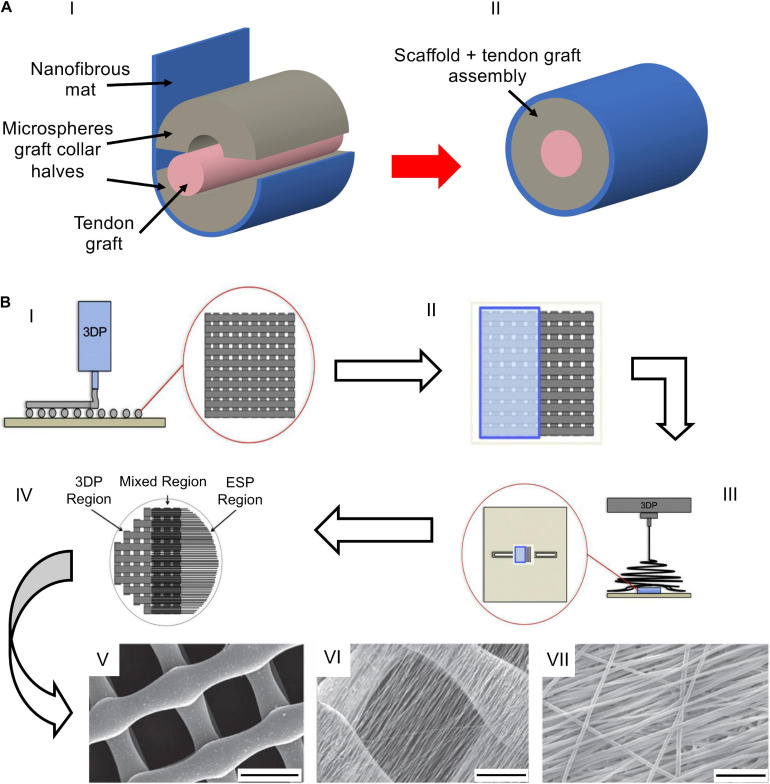
Examples of composite and 3D structures. **(A)** The composite scaffold adapted from Spalazzi et al. (2008): (AI) bovine patellar tendon (pink) embedded in a microspheres graft collar (gray) wrapped with a nanofibrous mat (blue); (AII) complete assembly. **(B)** Fabrication process adapted from Criscenti et al. (2016) (reproduced with permission. Copyright 2016, IOP Publishing Ltd): (BI) Fabrication of the AM PCL scaffold; (BII) covering of the AM scaffold with a paper foil; (BIII) electrospinning of PLGA on the PCL grid; (BIV) resulting mat with 3 regions (AM, mixed, ESP); (BV–BVII) SEM images of the regions [(BV) AM region: scale bar = 500 μm; (BVI) mixed region: scale bar = 200 μm; (BVII) ESP region: scale bar = 20 μm].

Until now, the works have tried to improve the healing and repair of the injured junctions, using simplified mats. A groundbreaking improvement in the field was performed when researchers have started to develop junction-inspired hierarchical nanofibrous scaffolds, reproducing the tissue gradients along both their length and thickness. A pioneering study in this field was done by Spalazzi et al., aiming to induce the enthesis fibrocartilage expression on ACL bovine specimens. They covered a circular scaffold of sintered nanospheres with an aligned nanofiber mat of PLGA (Spalazzi et al., 2008; Figure 8A). The shrinkage of the simple nanofiber mesh revealed no significant differences between the control group and the scaffold one. Conversely the contraction of the mat+graft collar led to an increased matrix density. After 2 weeks, the control group kept the characteristic crimp while the mat+graft collar retained its dense matrix pattern with a high cellularity. In particular, they studied how the shrinkage of nanofibers could induce a tendon matrix collagen distribution, cellularity, proteoglycan amount, and gene expression over 2 weeks. They found an upregulated expression of fibrocartilage-related markers such as Type II collagen, ACAN, and transforming growth factor-b3 (TGF-b3) (Spalazzi et al., 2008). In the previously mentioned work of Samavedi et al. (2014) they designed also a 3D fascicle with different regions in fiber orientation, diameter, and mechanical and chemical properties. They cut the electrospun mats into pieces that were rolled around a guide, obtaining a bundle with different nanofiber organizations (i.e., extremities = random PLGA; center = axially aligned). The tensile test showed that PCL10.5–PLGA13 had higher values of stiffness compared to the PCL7.5–PLGA13 ones (Samavedi et al., 2014). In a later study, Criscenti et al. (2016) fabricated an innovative triphasic scaffold for ligament-to-bone regeneration, combining an additive manufacturing (AM) reticular structure and an electrospun mat of aligned nanofibers Figure 8B). Firstly, they produced the AM bone-inspired structure, and then they partially covered the scaffold with an electrospun aligned nanofiber membrane of PCL (tendon side), obtained with a gap collector strategy. In this way, three different regions were obtained (tendon, bone, and the interface side). The biomechanical tests revealed that the electrospun region (ESP) had a failure stress of 5.21 ± 1.11 MPa and a stiffness of 88.9 ± 15.1 MPa, the AM region had a failure stress of 1.62 ± 0.27 MPa and a stiffness of 43.6 ± 8.1 MPa while the triphasic region (mixed) had a failure stress of 2.57 ± 0.51 MPa and a stiffness of 50.6 ± 10.5 MPa. The hBMMSCs revealed a higher proliferation in the ESP region, whereas an osteogenic differentiation was found to be higher in the AM side. Ligamentogenesis was found to be higher in the triphasic region (Criscenti et al., 2016). This methodology opened the way, for the first time in the interfacial tissue engineering, to match together electrospinning and AM to reproduce the tissue gradients of the enthesis. Lin et al. (2017) realized a structure with a random-to-aligned nanofibrous gradient to mimic the fiber arrangement at the ligament–bone insertion site. A solution of PCL was electrospun on a dual motor gap collector. The collector was composed of a pair of steel cones (random nanofibers) with a gap between them (aligned nanofibers in the gap). At the end of the process, a central bundle of aligned nanofibers, with two conical random extremities, was produced. The mechanical analysis revealed higher values of failure stress for the aligned region compared to the random one. The cellular tests, carried out with hBMMSCs, revealed that cells resulted elongated along the nanofibers’ direction (aligned region), whereas they were randomly oriented inside the random region. The gene expression assay showed that the tenogenic markers’ expression was significantly higher in the aligned region, while the osteogenic markers’ expression was more intense in the random region (Lin et al., 2017). He et al. (2017) also moved their attention to ligament–bone enthesis, realizing a microfiber-reinforced mat. The mat was produced with the same setup of the previous work (Figure 6A) to obtain a random and aligned structure. The two syringes were loaded with PLGA and nHap-BMP2-PLGA. A layer of PLGA was spun on the drum obtaining aligned nanofibers, then the syringe was stopped and PLLA microfibers were circumferentially fixed around the mandrel. A new layer of pure aligned PLGA nanofibers was deposited until the microfibers were fully covered (tendon side). The PLGA syringe was stopped, and the nHap-BMP2-PLGA one was started to cover the mat with random nanofibers (bone side). The resulting meshes were seeded with MC3T3-E1, and cell morphology, proliferation, and differentiation were evaluated after 10 days. The results showed an aligned cytoskeleton only in the aligned region and a high proliferation at 6 days. The differentiation assay evinced that nHap-BMP2 had a much more effect on the osteogenic differentiation than the fiber orientation. The nanofiber mats were further wrapped up to form 3D gradient scaffolds, which were mechanically tested. The mats of 2 or 5 cm width showed a yield force and a failure force that were improved with the increasing of the width. A preliminary animal study was performed on 10 rabbits ACL and histologically characterized. After 3 months of implantation, the histological evaluation showed the formation of new collagen fibers, which were aligned in the direction of the microfibers. The amount of the new formed fibers decreased with the gradient of nHap-BMP2. Moreover, a revascularization was observed with a higher amount of blood vessels at the region with higher concentration of nHap-BMP2. Conversely, few blood vessels were found in the region with a lower nHap-BMP2 concentration (He et al., 2017). More recently, Olvera et al. (2020) continued their studies on the ligament enthesis, realizing an electrospun microfibrous scaffold functionalized with extracellular matrix (ECM) components. To obtain the fibers, the electrospinning setup was composed by a syringe pump, loaded with PCL, and a high-speed rotating drum collector. After electrospinning, the mats were wrapped into bundles. The mechanical test displayed a stiffness of 121.5 ± 3.8 MPa, a yield stress of 6.3 ± 0.09 MPa, and yield strain of ∼10%. Collagen Type I (Col-I), cartilage ECM (C-ECM), and ligament ECM (C-ECM) were immobilized on the scaffold by a physical adsorption or a covalent conjugation. For the gene expression, the scaffolds were seeded with MSCs and analyzed at days 0 and 10. For the cell viability tests, they were analyzed after 1 day of cell culture. Moreover, part of the scaffold (C-ECM region), was coated with a bone-like apatite by soaking that region in a 10-SBF solution. After 1 day of cell culture, cells displayed different forms according to the region in which they were seeded. The cells on the L-ECM region had an elongated form. The cells on the C-ECM region showed a more rounded shape while the ones seeded on the collagen Type I region had a mix of elongated and rounded shape. The physical incorporation of every kind of protein (L-ECM or C-ECM or Col-I) promoted the Col1a1 and Col3a1 expression in pBMMSCs. In particular, the L-ECM physical incorporation promoted also the expression of the specific ligamentous marker Tnmd, showing a better ligamentogenesis influence than the Col-I functionalization. Conversely, the covalent immobilization of C-ECM resulted in the highest SRY-Box 9 expression level, fundamental for the cartilage formation. The differentiation was further investigated in the presence of growth factors. The scaffolds were kept in the culture media augmented with CTGF or TGF-β3. The addition of CTGF increased the expression of Col1a1, Col3a1, and Tnmd in the L-ECM membrane and in the Col-I scaffolds. L-ECM confirmed the best performances in the enhancement of the ligamentogenesis. The chemically immobilized C-ECM had the higher values of SOX9 expression level. TGF-β3 showed the highest levels of cartilage-specific genes like cartilage oligomeric matrix protein (COMP), SRY-Box 9, and ACAN. After assessing the influence of L-ECM and C-ECM on singular scaffolds, an aligned triphasic scaffold was produced. The scaffold had three different regions: immobilized L-ECM region, C-ECM region, and C-ECM + Hap region. The immobilization was achieved, incubating the scaffolds in different ECM solutions with the chemical immobilization method. The cell morphology followed the trends of the singular scaffolds previously tested. The cell differentiation after 10 days of culture exhibited the highest values of Tnmd in both the L-ECM and C-ECM regions. Finally, the mineralized region of C-ECM + Hap displayed the highest levels of COMP, ACAN, OPN, and Col10a1 expression (Olvera et al., 2020).

## Conclusion and Future Perspective

The regeneration of the musculoskeletal junctions represents one of the biggest challenges for the tissue engineering. Various techniques have been used trying to improve this field, and, among these, electrospinning has proved to be one of the most promising. Over the years, and in particular in the last decades, different materials and designs have been used, obtaining encouraging results. Starting from simple mats, gradually the scaffolds assumed an increasing hierarchical complexity, enriching their bioactivity and cell differentiation by using drugs, nanoparticles, and gradients of mineralization. Despite the encouraging outcomes, several improvements concerning the scaffolds’ multiscale morphology and mechanical properties are needed, making them still inadequate for an implantation in human patients. In fact so far, no complex hierarchical structures have been used able to mimic the whole levels of aggregation of the enthesis and the MTJ. Moreover, a lot of work has to be done at the enthesis side, to guarantee an effective bone-inspired anchoring to the surrounding tissues. At the MTJ instead, the interdigitated structure of this interface is still an open challenge. A possible promising solution could be offered by improving the integration between AM, bioprinting, and electrospinning. This will certainly open the way to scaffolds that come closer and closer to mimicking the performance of natural tissues by combining mineralization gradients and mechanical properties, making them more ideal environments for cell proliferation and differentiation.

## Author Contributions

AS and LC conceptualized the study. GM with the help of AS made the literature search and analyzed the target manuscripts. AS and GM wrote the original draft and prepared the figures with help from CG. LC, AS, and AZ reviewed and edited the draft. AS, LC, and AZ supervised the work. All authors listed have made a substantial, direct and intellectual contribution to the work, and approved it for publication.

## Conflict of Interest

AS, LC, and AZ hold an international patent on a related invention (“Hierarchical multiscale electrospun scaffold for the regeneration and/or replacement of the tendinous/ligamentous tissue and a method for its production” WO2018/229615 A1 of 20 December 2018). The authors declare that the research was conducted in the absence of any commercial or financial relationships that could be construed as a potential conflict of interest.

## Supplementary Material

Supplementary Table 1 Mechanical properties of electrospun scaffolds for interfacial tissue engineering.
