# Supplementary material for: Tissue Engineering for the Insertions of Tendons and Ligaments: An Overview of Electrospun Biomaterials and Structures
Source: Front Bioeng Biotechnol. 2021 Mar 2;9:645544. doi: 10.3389/fbioe.2021.645544 (PMC7961092; doi:10.3389/fbioe.2021.645544)
Supplement: Supplementary Table 1 — Mechanical properties of electrospun scaffolds for interfacial tissue engineering. [file Table_1.docx]

Supplementary Table 1| Mechanical properties of electrospun scaffolds for interfacial tissue engineering.

| **Test type** | **Stiffness**  **(N/mm)** | **Load to Failure**  **(N)** | **Failure Stress**  **(MPa)** | **Failure Strain**  **(%)** | | **Applications** | **References** |
| --- | --- | --- | --- | --- | --- | --- | --- |
| Uniaxial Tensile Test | From mineralized 120  down to 30 | - | - | 0% - 2% at 0.3 MPa  1% - 4% at 1.4 MPa   0% - 7% at 2.2 MPa | | Tendon-Bone | (Li et al., 2009) |
| Uniaxial Tensile Test | ~ 75(Aligned)   ~ 30(Random) | - | ~ 45 (Aligned)  ~ 20 (Random) | - | | Tendon-Bone | (Xie et al., 2010) |
| Uniaxial Tensile Test | 4.490 ± 1.604 (PCL Side) 20.06 ± 7.773 (Center) 27.62 ± 6.063 (PLLA Side) 7.339 ± 2.131 (Whole Scaffold) 0.2789 ± 0.1509 (Native MTJ) | - | 1.069 ± 0.2713 (PCL Side) 2.384 ± 0.5987 (Center) 3.741 ± 0.8486 (PLLA Side) 0.5058 ± 0.2130 (Whole) 0.1478 ± 0.01631 (Native MTJ) | 130.4 ± 44.56 (PCL Side) 42.79 ± 17.75 (Center) 35.33 ± 8.964 (PLLA Side) 18.49 ± 8.210 (Whole) 122.4 ± 19.18 (Native MTJ) | | Tendon-Bone | (Ladd et al., 2011) |
| Uniaxial Tensile Test | * nHAP-PCL = 0.64 ± 0.04  GRAD = 0.58 ± 0.09  PEUUR2000 = 0.22 ± 0.03  ** nHAP-PCL = 2.4 ± 0.23  GRAD = 0.55 ± 0.01   PEUUR2000 = 0.23 ± 0.04   *Unmineralized  **Mineralized | - | * nHAP-PCL = 0.2   GRAD = 0.5  PEUUR2000 = 0.6  ** nHAP-PCL = 0.45 GRAD = 0.4 PEUUR2000 = 0.6  *Unmineralized **Mineralized | * nHAP-PCL = 45 ± 5   GRAD = 125 ± 25  PEUUR2000 = 165 ± 5  ** nHAP-PCL = 75 ± 25  GRAD = 125  PEUUR2000 = 250  *Unmineralized **Mineralized | | Ligament-Bone | (Samavedi et al., 2011) |
| Uniaxial Tensile Test | ~560 (mineralization: 6 h)   ~ 350 (mineralization: 2 h)  ~ 250 (mineralization: 0 h) (Obtained by graphic) | - | (Yield Stress) ~ 5 (mineralization: 6 h)  ~ 6 (mineralization: 2 h)  ~ 7 (mineralization: 0 h)  (Obtained by graphic) | - | | Tendon-Bone | (Liu et al., 2011) |
| Uniaxial Tensile Test | Scaffold Reattachment  *  1.3 ± 0.3 2.4 ± 0.3 **  6.1 ± 2.2 7.3 ± 2.6  ***  12.8 ± 3.1 11.0 ± 3.1 ****  12.8 ± 3.1 10.2 ± 1.4  *0 Weeks **4 Weeks ***8 Weeks ****16 Weeks | Scaffold Reattachment  * 5.4 ± 2.5 16.9 ± 6.0 ** 28.1 ± 12.0 61.7 ± 9.5 *** 71.7 ± 12.0 68.3 ± 21.5 **** 75.3 ± 18.7 69.4 ± 14.9  *0 Weeks **4 Weeks ***8 Weeks ****16 Weeks | - | - | | Tendon-Bone | (Inui et al., 2012) |
| Uniaxial Tensile Test | * 1650 ± 150 (Uniform circular)  1390 ± 100 (Non uniform)  2250 ± 250(Uniform ellipsoidal) ** 2490 ± 180 (Thick-platelet)  1260 ± 230 (Thick-conformal)  1570 ± 170 (Thin Conformal)  *Pristine PLGA **Mineralized PLGA | - | * 56 ± 3 (Uniform circular)  89 ± 7 (Non uniform)  145 ± 12 (Uniform ellipsoidal) **  30 ± 4 (Thick-platelet) 67 ± 0 (Thick-conformal) 136 ± 11 (Thin-conformal)  *Pristine PLGA **Mineralized PLGA | | * 150 ± 25 (Uniform circular)  122 ± 7 (Non uniform) 81 ± 7 (Uniform ellipsoidal)  *Pristine PLGA | Tendon-Bone | (Kolluru et al., 2013) |
| Monotonic Tensile Test | * Random (PLGA) 27.8 ± 7.9 Aligned (PCL) 6.8 ± 3.7  ** Random (PLGA) 23.8 ± 4.4 Aligned (PCL) 9.9 ±2.8   *PCL7.5-PLGA13 **PCL10.5-PLGA13 | - | * Random (PLGA) 25 ± 9  Aligned (PCL) 50 ± 14 ** Random (PLGA) 24 ± 2 Aligned (PCL) 41 ± 12  *PCL7.5-PLGA13 **PCL10.5-PLGA13 | - | | Ligament-Bone | (Samavedi et al., 2014) |
| Uniaxial Tensile Test | 3100 | - | **-** | - | | Tendon-Bone | (Lipner et al., 2014) |
| Uniaxial Tensile Test | *  5.4 ± 0.6 (Controls)  5.7 ± 0.7 (PLGA)  6.1 ± 0.6 (bFGF–PLGA)  **  7.9 ± 0.8 (Controls)   9.4 ± 0.8 (PLGA)  9.7 ± 0.5 (bFGF–PLGA)  ***  13.0 ± 0.7 (Controls)   13.7 ± 0.7 (PLGA)  14.9 ± 0.3 (bFGF–PLGA)  *2 Weeks **4 Weeks ***8 Weeks | * 8.3 ± 0.6 (Controls) 8.7 ± 0.9 (PLGA)  9.1 ± 0.9 (bFGF–PLGA)  ** 18.2 ± 0.9 (Controls)   20.7±1.6 (PLGA)  21.4±1.3 (bFGF–PLGA) *** 27.1 ± 1.2 (Controls)  28.4 ± 1.2 (PLGA)  32.7 ± 1.0 (bFGF–PLGA)  *2 Weeks **4 Weeks ***8 Weeks | * 1.00 ± 0.02 (Controls)  1.00 ± 0.02 (PLGA)  1.02±0.02 (bFGF–PLGA)  ** 1.30±0.08 (Controls)  1.32 ± 0.06 (PLGA)  1.33 ± 0.05 (bFGF–PLGA)  *** 1.65 ± 0.09 (Controls)  1.62 ± 0.03 (PLGA)  1.82 ± 0.03 (bFGF–PLGA)  *2 Weeks **4 Weeks ***8 Weeks | - | | Tendon-Bone | (Zhao et al., 2014) |
| Uniaxial Tensile Test | * ~ 5 (Control) ~ 5 (PLLA) ~ 5.5 (gelatin-PLLA) ** ~ 8.5 (Control) ~ 8.5 (PLLA) ~ 9.5 (gelatin-PLLA) *** ~ 13 (Control) ~ 13.5 (PLLA) ~ 14.5 (gelatin-PLLA)  *2 Weeks *4 Weeks *8 Weeks | * ~ 8 (Control) ~ 8 (PLLA) ~ 8 (gelatin-PLLA) ** ~ 19 (Control) ~ 20 (PLLA) ~ 21 (gelatin-PLLA) *** ~ 27 (Control) ~ 28 (PLLA) ~ 31 (gelatin-PLLA)  *2 Weeks *4 Weeks *8 Weeks | * ~ 1.0 (Control) ~ 1.0 (PLLA) ~ 1.0 (gelatin-PLLA) ** ~ 1.3 (Control) ~ 1.3 (PLLA) ~ 1.4 (gelatin-PLLA) *** ~ 1.7 (Control) ~ 1.6 (PLLA) ~ 1.8 (gelatin-PLLA)  *2 Weeks *4 Weeks *8 Weeks | - | | Tendon-Bone | (Zhao et al., 2015) |
| Uniaxial Tensile Test | *  ~ 8.5 (PCL/nHAp/Col) ~ 7 (PCL)  ** 15.2 ± 1.4 (PCL/nHAp/Col) 10.2 ± 1.3 (PCL)  *4 weeks **8 weeks | *  ~ 28 (PCL/nHAp/Col) ~ 25 (PCL)  ** 58.4 ± 4.1 (PCL/nHAp/Col) 39.9 ± 3.4 (PCL)  *4 weeks **8 weeks | - | - | | Tendon-Bone | (Han et al., 2015) |
| Uniaxial Tensile Test | *  ~ 7 (Suture)  ~ 7.5 (Acellular)  ~ 6.5 (Cellular)  ~ 4 (Cellular-BMP2)  **  ~ 13 (Suture)  ~ 8 (Acellular)  ~ 10 (Cellular)  ~ 7.5 (Cellular-BMP2)  *28 days **56 days | *  21 ± 5.5 (Suture) 25 ± 5.9 (Acellular) 25.7 ± 9.4 (Cellular) 19.7 ± 5.9 (Cellular-BMP2)  ** 32.3 ± 4.9 (Suture) 31.1 ± 9.4 (Acellular) 31.7 ± 10 (Cellular) 32.6 ± 6.6 (Cellular-BMP2)  *28 days **56 days | *  ~ 1.15 (Suture)  ~ 1.4 (Acellular)  ~ 1.4 (Cellular)  ~ 0.75 (Cellular-BMP2)  **  ~ 2 (Suture)  ~ 1.4 (Acellular)  ~ 2 (Cellular)  ~ 1.6 (Cellular-BMP2)  *28 days **56 days | *  34.14 ± 14 (Suture) 39 ± 29 (Acellular) 42 ± 11 (Cellular)  42 ± 16 (Cellular-BMP2)  **  31.8 ± 8 (Suture) 34 ± 7 (Acellular) 38 ± 11 (Cellular) 50 ± 20 (Cellular-BMP2)  *28 days **56 days | | Tendon-Bone | (Lipner et al., 2015) |
| Uniaxial Tensile Test | * ~ 6.5 (Control) ~ 7.5 (SF) ** ~ 9.5 (Control) ~ 14 (SF)  *6 Weeks **12 Weeks | * ~ 31 (Control) ~ 40 (SF) ** ~ 51 (Control) ~ 67 (SF)  *6 Weeks **12 Weeks | - | - | | Tendon-Bone | (Zhi et al., 2016) |
| Uniaxial Tensile Test | * 82.8 ± 11.6 **  33.6 ± 14.8   *Aligned Region **Random region | - | * 38.7 ± 6.2 ** 6.3 ± 3.2  *Aligned Region **Random region | * 132.5 ± 26.4 ** 61.5 ± 13.3  *Aligned Region **Random region | | Ligament-Bone | (Lin et al., 2017) |
| Uniaxial Tensile Test | * 43.6 ± 8.1 ** 50.6 ± 10.5 *** 88.9 ± 15.1  *AM **Mixed ***ESP | - | * 1.62 ± 0.27 ** 2.57 ± 0.51 *** 5.21 ± 1.11  *AM **Mixed ***ESP | * 4.81 ± 0.69 ** 6.71 ± 0.31 *** 22.1 ± 3.2  *AM **Mixed ***ESP | | Ligament-Bone | (Criscenti et al., 2016) |
| Uniaxial Tensile Test | - | #* ~20  ##* ~ 20  #** ~ 41.4 (Average) (Range: 37.4–44.5) ##** ~ 28.3 (Average) (Range: 26.1–30.5)  *8 Weeks **16 Weeks # Bolt+mat ## Control | - | - | | Tendon-Bone | (Chou et al., 2016) |
| Uniaxial Tensile Test | * ~ 1.8 (BPUR 10) ~ 7.5 (Transition) ~ 12.3 (BPUR 50) ** ~ 2 (BPUR 10) ~ 11 (Transition) ~ 17 (BPUR 50)  * Random Gradient ** Aligned Gradient | - | * ~ 4.5 (BPUR 10) ~ 3 (Transition) ~ 3.5 (BPUR 50) ** ~ 4 (BPUR 10) ~ 3.8 (Transition) ~ 4 (BPUR 50)  * Random Gradient ** Aligned Gradient | * ~ 210 (BPUR 10) ~ 160 (Transition) ~ 145 (BPUR 50) ** ~ 190 (BPUR 10) ~ 100 (Transition) ~ 75 (BPUR 50)  * Random Gradient ** Aligned Gradient | | Tendon-Bone | (Kishan et al., 2017) |
| Uniaxial Tensile Test | - | * Nanofibrous Scaffolds 40N  Micronanofibrous Scaffolds 200N ** Nanofibrous Scaffolds 100N Micronanofibrous Scaffolds 500N  *Film Width 2 cm **Film Width 5cm | - | - | | Ligament-Bone | (He et al., 2017) |
| Uniaxial Tensile Test | * ~ 11 (Control) ~ 11 (SFM) ~ 10.5 (BFM) ** ~ 13 (Control) ~ 13 (SFM) ~ 15 (BFM) *** ~ 13 (Control) ~ 16 (SFM) ~ 23 (BFM)  * 4 weeks ** 8 weeks *** 12 weeks | * ~ 85 (Control) ~ 100 (SFM) ~ 75 (BFM) ** ~ 100 (Control) ~ 140 (SFM) ~ 160 (BFM) *** ~ 120 (Control) ~ 150 (SFM) ~ 180 (BFM)  * 4 weeks ** 8 weeks *** 12 weeks | * ~ 3 (Control) ~ 3 (SFM) ~ 2.5 (BFM) ** ~ 3 (Control) ~ 3.5 (SFM) ~ 4 (BFM) *** ~ 3.5 (Control) ~ 3.8 (SFM) ~ 4.7 (BFM)  * 4 weeks ** 8 weeks *** 12 weeks | - | | Tendon-Bone | (Li et al., 2017) |
| Uniaxial Tensile Test | * 21 (PCL600nm) 30 (PCL1000nm) 24 (PLA) 15 (PCL aligned) 32 (PLLA out-PCL in) 60 (PCL out-PLLA in) ** 20 (PCL 600nm) 36 (PCL 1000nm) 43 (PLA) 63 (PCL aligned) 30 (PLLA out-PCL in) 38 (PCL out-PLLA in)  * Dry  ** Wet | - | - | - | | Tendon-Bone | (Baudequin et al., 2017) |
| Uniaxial Tensile Test | * 9.9 ± 1.9 (ARM)  9.3 ± 1.4 (RM) 5.8 ± 1.3 (Control) ** 21.5 ± 3.5 (ARM)   15.6 ± 1.6 (RM) 10.0 ± 1.1 (Control)  *6 Weeks **12 Weeks | * 43.9 ± 7.5 (ARM)  41.4 ± 5.7 (RM)  25.3 ± 5.9 (Control) **  83.2 ± 12.4 (ARM)  66.2 ± 6.6 (RM) 50.6 ± 3.5 (Control)  *6 Weeks **12 Weeks | - | - | | Tendon-Bone | (Cai et al., 2018) |
| Uniaxial Tensile Test | 215.5 (nHap-PCL/CS) 180 (PCL/CS) | 250 (nHap-PCL/CS) 195 (PCL/CS) | - | - | | Tendon-Bone | (Wu et al., 2018) |
| Uniaxial Tensile Test | * ~ 5 (PCL) ~ 7.5 (B@P) ~ 7 (S + B@P) ** 10.8 (PCL) 13.9 (B@P) 19.5 (S + B@P)  * 6 weeks ** 12 weeks | * ~ 18 (PCL) ~ 30 (B@P) ~ 33 (S + B@P) ** 42.7 (PCL) 63.5 (B@P) 79.9 (S + B@P)  * 6 weeks ** 12 weeks | - | - | | Tendon-Bone | (Han et al., 2019) |
| Uniaxial Tensile Test | * 17 (Obtained by graphic)  ***** Li+@MSN/PEUU | - | * 12 (Obtained by graphic)  ***** Li+@MSN/PEUU | * 70% (Obtained by graphic)  ***** Li+@MSN/PEUU | | Tendon-Bone | (Huang et al., 2020) |
| Uniaxial Tensile Test | * ~ 5.5 (Control) ~ 5.5 (PCL) ~ 6.5 (Melatonin-PCL) ** ~ 7.5 (Control) ~ 7.5 (PCL) ~ 10 (Melatonin-PCL) *** ~ 11 (Control) ~ 13.5 (PCL) ~ 15.4 (Melatonin-PCL)  * 2 weeks ** 4 weeks *** 8 weeks | * ~ 10 (Control) ~ 10 (PCL) ~ 10 (Melatonin-PCL) ** ~ 15 (Control) ~ 15 (PCL) ~ 20 (Melatonin-PCL) *** ~ 20 (Control) ~ 25 (PCL) ~ 33 (Melatonin-PCL)  * 2 weeks ** 4 weeks *** 8 weeks | * ~ 1.2 (Control) ~ 1.2 (PCL) ~ 1 (Melatonin-PCL) ** ~ 1.6 (Control) ~ 1.5 (PCL) ~ 2.3 (Melatonin-PCL) *** ~ 2.1 (Control) ~ 2.7 (PCL) ~ 3.3 (Melatonin-PCL)  * 2 weeks ** 4 weeks *** 8 weeks | - | | Tendon-Bone | (Song et al., 2019) |
| Uniaxial Tensile Test | - | * ~ 9 (Repair only) ~ 9.3 (PCL) ~ 9.7 (KGN-PCL) ** 15.3 ± 0.7 (Repair only) 19.4 ± 1.4 (PCL) 22.1 ± 0.5 (KGN-PCL) *** 20.7 ± 0.5 (Repair only) 25.5 ± 0.2 (PCL) 29.7 ± 1.6 (KGN-PCL)  * 2 weeks ** 4 weeks *** 8 weeks | - | - | | Tendon-Bone | (Zhu et al., 2019) |
| Uniaxial Tensile Test | * ~ 5.5 (Control) ~ 5.7 (PLGA) ~ 6.3 (PLGA/GO) ** ~ 8 (Control) ~ 8.2 (PLGA) ~ 9.8 (PLGA/GO) *** ~ 10 (Control) ~ 11.7 (PLGA) ~ 12.5 (PLGA/GO)  * 4 weeks ** 8 weeks *** 12 weeks | * ~ 60 (Control) ~ 60 (PLGA) ~ 70 (PLGA/GO) ** ~ 90 (Control) ~ 95 (PLGA) ~ 115 (PLGA/GO) *** ~ 125 (Control) ~ 130 (PLGA) ~ 150 (PLGA/GO)  * 4 weeks ** 8 weeks *** 12 weeks | * ~ 4.5 (Control) ~ 4.5 (PLGA) ~ 5.8 (PLGA/GO) ** ~ 5.3 (Control) ~ 5.8 (PLGA) ~ 6.5 (PLGA/GO) *** ~ 6.3(Control) ~ 6.3 (PLGA) ~ 7.5 (PLGA/GO)  * 4 weeks ** 8 weeks *** 12 weeks | - | | Tendon-Bone | (Su et al., 2019) |
| Uniaxial Tensile Test | 7.74 ± 0.91 (CS–g–PCL)  8.06 ± 0.92 (TGF– β3–CS–g–PCL)  12.46 ± 0.55 (Native enthesis) | 15.7 ± 9.4 (CS–g–PCL)  23.2 ± 9.4 (TGF– β3–CS–g–PCL)  29.6 ± 7.9 (Native enthesis) | - | - | | Tendon-Bone | (Reifenrath et al., 2020) |
